# Targeting VDAC1 to protect against mitochondria-linked cell death pathways: apoptosis, pyroptosis, ferroptosis, and associated diseases

**DOI:** 10.1007/s10495-025-02217-7

**Published:** 2026-04-11

**Authors:** A. Shteinfer-Kuzmine, A. Karunanithi Nivedita, M. Santhanam, S. Trishna, R. W. Swerdlow, J. Pan, V. Shoshan-Barmatz

**Affiliations:** 1 National Institute for Biotechnology in the Negev, Ben-Gurion University of the Negev, 84105 Beer-Sheva, Israel; 2 Department of Life Sciences, Ben-Gurion University of the Negev, 84105 Beer-Sheva, Israel; 3 University of Kansas Alzheimer’s Disease Research Center, 3901 Rainbow Boulevard, Kansas City, KS 66160 USA; 4 X-Tosis, 3501 S Main Street, Suite 1, Gainesville, FL 32601 USA

**Keywords:** Apoptosis, Ferroptosis, Inflammation, Mitochondria, Pyroptosis, Oligomerization, VDAC1

## Abstract

The pathways of programmed cell death (PCD), including apoptosis, pyroptosis, and ferroptosis, are interconnected. They can be activated simultaneously within tissues or cell lines and are often associated with various diseases. Thus, identifying a common player and inhibitor targeting several PCD types is essential. Here, we show that overexpression and oligomerization of the mitochondrial gatekeeper voltage-dependent anion channel 1 (VDAC1) is involved in apoptosis, pyroptosis, and ferroptosis, and specific VDAC1 oligomerization inhibitors, VBIT-4 and VBIT-12, prevented multiple forms of PCD triggered by various stimuli. In addition, they mitigated mitochondrial dysfunction, reduced reactive oxygen species production and intracellular Ca^2^⁺ levels, preserved mitochondrial-associated hexokinase, and inhibited assembly/activation of the NLRP3 inflammasome. In Alzheimer’s disease and inflammatory bowel disease mouse models, VBIT-4 and VBIT-12, respectively, protected against apoptosis, pyroptosis, ferroptosis, and disease-associated pathologies. Thus, we show that VDAC1 oligomerization represents a prime target for VBIT-4 and VBIT-12 that can simultaneously inhibit various PCD forms and diseases associated with enhanced PCD and/or inflammation.

**Supplementary Information:**

The online version contains supplementary material available at 10.1007/s10495-025-02217-7.

## Introduction

Cell death has physiological and pathological functions. Programmed cell death (PCD), encompasses a variety of processes such as necroptosis, apoptosis, autophagy, pyroptosis, and ferroptosis [1]. These PCD forms are not completely separated and can coexist within the same cell or tissue. Each can be activated via multiple pathways simultaneously, leading to a complex cross-regulation of cell death types [2, 3]. The mechanisms that regulate these pathways are tightly connected and can cross-regulate each other, resulting in a dynamic system of cellular death [1]. Thus, dysregulation of PCD has been linked to various human pathologies, including diabetes, autoimmune inflammatory conditions, and neurodegenerative disorders [4–12].

Apoptosis is triggered through two primary signaling routes: the extrinsic and intrinsic pathways, both of which culminate in the activation of caspases [3]. The extrinsic pathway is initiated when external molecules bind to death receptors on the cell surface, such as Fas/CD95 or tumor necrosis factor resulting in the activation of pro-caspase-8, which in turn activates caspases-3/7 and triggers cell death [13].

On the other hand, the intrinsic pathway, which is dependent on mitochondrial signals, is activated by internal stressors such as DNA damage, elevated intracellular Ca^2+^, increased reactive oxygen species (ROS) levels, certain chemotherapy drugs [14], and by overexpressing mitochondrial VDAC1 [15]. These stimuli prompt the mitochondria to release pro-apoptotic factors, including cytochrome* c* (Cyto *c*), SMAC, endonuclease G (Endo-G), and apoptosis-inducing factor (AIF). Cyto *c* then associates with apoptotic protease activating factor 1 (Apaf-1), and together with inactive pro-caspase-9, forms the apoptosome, resulting in pro-caspase-9 activation that initiates caspase-3/7, and finally apoptosis.

Several mechanisms have been proposed to explain how the apoptotic initiators cross the outer mitochondrial membrane (OMM). These include opening of the permeability transition pore (PTP) [16], formation of large channels by oligomerized BAX and/or BAK proteins [17], and involvement of VDAC1 oligomers [18, 19] (presented below).

Pyroptosis is an inflammatory form of programmed cell death that involves the activation of inflammasomes, particularly the NOD-like receptor protein 3 (NLRP3) inflammasome [20]. It is characterized by the formation of a gasdermin (GSDM)-mediated pore in the plasma and mitochondrial membranes [6, 7]. Caspase-1, activated via the inflammasome, targets gasdermin D (GSDMD) [21], while caspase-3, activated through the apoptosome, cleaves gasdermin E (GSDME) also known as DFNA5 (deafness, autosomal dominant 5) [22, 23]. In both cases, the cleavage generates N-terminal fragments that oligomerize to form large pores in the cell membrane. For GSDMD, these pores enable the release of pro-inflammatory cytokines (e.g., IL-1β, IL-18), thereby driving inflammatory responses [6, 7].

The GSDME-derived N-terminal fragment forms pores in both the mitochondrial and plasma membranes, driving the transition from apoptosis to secondary necrosis [24].

Similar to apoptotic cells, pyroptotic and secondary necrotic cells display mitochondrial membrane depolarization, DNA fragmentation, and nuclear condensation, show positive TUNEL staining, and with the involvement of caspase-3, represent an intersection between apoptosis and GSDME-mediated pyroptosis/secondary necrosis. Pyroptosis-associated inflammation has been linked to various pathologies, including inflammatory diseases, cancers [25, 26], and central nervous system (CNS) disorders [6–8].

Ferroptosis is an iron-dependent type of PCD, characterized by the oxidation of polyunsaturated fatty acids (PUFAs) and the buildup of lipid peroxides and iron, with the mitochondria contributing in multiple roles to this process [27–29]. This PCD is also characterized by mitochondrial shrinkage and reduced cristae, along with increased ROS production and lipid peroxidation [30]. VDAC1 has been proposed to mediate ROS release from the IMS to the cytosol [31]. Ferroptosis is associated with a decrease in intracellular glutathione (GSH) and diminished activity of glutathione peroxidase 4 (GPX4), which converts GSH to GSSG, an antioxidant system that helps mitigate ROS levels and suppress ferroptosis [5, 32]. Ferroptosis contributes to the development and progression of various diseases, such as cancer, stroke, brain injury, ischemia–reperfusion injury, kidney failure, neurodegeneration, and T-cell immunity [4, 5, 9]. Inhibiting it by regulating GPX4 activity, trapping ROS, using ferroptosis-specific inhibitors, and chelating iron provides an intriguing opportunity for potential treatments of these diseases [33].

The extensive crosstalk among different types of PCD led to the concept of PANoptosis, which represents a coordinated mechanism incorporating elements of pyroptosis, apoptosis, and/or necroptosis [34, 35]. PANoptosis has been implicated in the pathogenesis of various diseases, including neurodegenerative disorders, inflammation, cancer, atherosclerosis, and autoimmune diseases [34, 36, 37].

In this work, we demonstrated the role of the mitochondria and their gatekeeper, VDAC1, in apoptosis, pyroptosis, and ferroptosis. Mitochondria serve as central regulators of various cellular processes, including metabolism, apoptosis, cell-cycle control, proliferation, differentiation, epigenetics, immune signaling, and aging [38]. VDAC1 plays a pivotal role as a mitochondrial governor, forming a channel in the OMM that facilitates the transport of ions, nucleotides, and various metabolites. It additionally contributes to mitochondrial apoptosis [18, 19, 39–42]. VDAC1 interacts with about 150 other proteins, thereby coordinating mitochondrial activity with broader cellular processes. [41, 42]. Our studies have demonstrated that the induction of apoptosis results in both overexpression and oligomerization of VDAC1 across various cell types and apoptosis inducers [10, 18, 19]. Accordingly, we proposed that apoptosis inducers, stress, and certain disease states trigger VDAC1 overexpression, shifting the equilibrium from monomeric to oligomeric states. This oligomerization leads to the formation of a large channel within the VDAC1 oligomer, enabling the release of pro-apoptotic proteins that drive the execution of apoptosis [10, 18, 19, 39–42]. Oligomerized VDAC1 also facilitates the release of mitochondrial DNA (mtDNA), which activates type-Ι interferon signaling and promotes inflammation [43, 44]. VDAC1 oligomerization has been shown to be essential for the assembly and activation of the NLRP3 inflammasome [45]. Our research, along with other studies, has highlighted that overexpression and oligomerization of VDAC1 are common across various diseases [10]. Consequently, targeting VDAC1 overexpression and/or oligomerization offers a promising treatment strategy.

In our quest to effectively prevent VDAC1 oligomerization, mitigate cell death, protect against mitochondrial dysfunction and mtDNA release, and suppress inflammation [43, 44, 46, 47], we developed two novel VDAC1-targeting molecules: VBIT-4 and VBIT-12. These molecules have demonstrated efficacy across diverse disease mouse models, including type-2 diabetes [48], lupus [43], colitis [47], Alzheimer’s disease (AD) [46], acute liver injury [49], spinal cord injury [50], and COVID-19 [51, 52]. These findings underscore VDAC1 as a critical regulator at the crossroads of metabolism, cell survival, apoptosis, and inflammation, linking mitochondrial dysfunction with a range of diseases. In this study, we demonstrated that VDAC1 overexpression and oligomerization play a direct role in multiple forms of PCD, including apoptosis, pyroptosis, and ferroptosis—all of which were inhibited by both VBIT-4 and VBIT-12. PCD activation contributes to diseases such as diabetes, autoimmune diseases, non-alcoholic steatohepatitis, and neurodegenerative diseases [4–10, 12]. Here, for the first time, we present compounds that inhibit the various PCD forms that are activated and associated with diseases such as AD and colitis. This is particularly important given that different PCD mechanisms are tightly connected and can cross-regulate each other, such that if one form of PCD is inhibited, other compensatory pathways may be activated in response [1–3]. Therefore, targeting a common feature across these pathways, as shown here, is a promising strategy for simultaneously targeting all forms of PCD.

### Materials and methods

Materials, dsDNA staining, cytochrome *c* release assay, protein extraction and quantification, gel electrophoresis and immunoblot analysis presented in the ***Supplementary Materials.***

#### Cell culture and treatments

HEK-293 (human embryonic kidney), HeLa (human, cervix adenocarcinoma), PC-3 (human, prostate adenocarcinoma), CT-26 (mouse colon carcinoma) C6 (rat glioma), A549 (human lung adenocarcinoma cells) and Hep-G2 (human, hepatocellular carcinoma) cell lines were from the American Type Culture Collection (ATCC) (Manassas, VA) and cultured according to ATCC guidelines. Cells were maintained in the recommended growth medium supplemented with 10% heat-inactivated fetal bovine serum (FBS), 100 U/mL penicillin, and 100 μg/mL streptomycin. Cultures were incubated at 37°C in a humidified atmosphere containing 5% CO₂ and were routinely screened for mycoplasma contamination. The treatment with the various reagents is described in the figure legends.

#### Cell death analyses

Cells were harvested, centrifuged (1500 g, 5 min), washed, and cell death was assessed using propidium iodide (PI) staining, followed by flow cytometry using an iCyt SY3200 Benchtop Cell Sorter/Analyzer (Sony Biotechnology Inc., San Jose, CA) and analyzed with EC800 software. Apoptosis was evaluated via dual staining with PI and Annexin V-FITC, performed according to the manufacturer’s instructions with minor modifications. Flow cytometry was used for sample analysis, with a minimum of 10,000 events collected and represented as dot plots.

#### VDAC1 silencing

VDAC1 silencing was performed by transfecting CT-26 cells or HeLa cells (150,000 cells/well, in 6-well plates, 40–60% confluence) with 50 nM or 75nM, respectively, of either non-targeting siRNA (si-NT) or siRNA targeting VDAC1 targeting both human and mouse VDAC1 (si-m/hVDAC1-B) using JetPRIME transfection reagent according to the manufacturers’ instructions. si-NT and si–h/mVDAC1-B were synthesized and obtained from Genepharma (Suzhou, China). si-NT, Sense**:** 5′GCAAACA**U**CCCA**G**AGG**U**AU3′, **Anti sense:** 5′ AUACC**U**CUG**G**GAUGUUUGC3’. si–m/hVDAC1-B, Sense: 5’ GAA**U**AGCAGCCAA**G**UA**U**CAGtt 3; Anti-sense: 5′CUGAUAC**U**UGGCU**G**CUAUUCtt 3′. The bold nucleotides were 2′-O-methyl modified.

#### mtDNA quantification

To quantify cytosolic mtDNA, CT-26 cells were treated with 100 mM H₂O₂ for 20 h, pelleted, and incubated for 10 min in a buffer composed of 150 mM NaCl, 50 mM HEPES, pH 7.4, and 25 µg/mL digitonin, followed by centrifugation at 16,000 × *g*, 20 min at 4°C. The supernatant was diluted 1:10 and used for qRT-PCR to detect mtDNA (D-Loop 3 region) using the specific primers; Forward: 5′- TCCTCCGTGAAACCAACAA-3′ Reverse: 5′-AGCGAGAAGGGGCATT-3′.

#### Plasmid preparation and transfection

The pEGFP-HK-I construct, encoding GFP fused to the C-terminus of HK-I, was generated as previously described [53]. Cells (1.5 × 10^5^) were seeded on coverslips in 6-well plates and transfected with pEGFP-HK-I using JetPRIME transfection reagent (Polyplus, llkirch-Graffenstaden, France), according to the manufacturer’s instructions. After 24 h, cells were pre-incubated with or without VBIT-4, followed by treatment with cisplatin (CP) for an additional 48 h, and then washed with PBS, fixed for 15 min with 4% paraformaldehyde, and rinsed with PBS prior to immunofluorescence and imaging by confocal microscopy (Olympus 1X81).

#### Immunofluorescence (IF) of cells

Cells (2 × 10^5^) were grown on coverslips and treated with the desired compound. After 48h, the cells were fixed for 15 min using 4% paraformaldehyde in PBS, followed by rinsing in PBS for 30 min. Cells were permeabilized with 0.3% Triton X-100 in PBS and blocked for 2 h with blocking buffer containing 10% normal goat serum and 1% fatty acid-free BSA in PBS. Subsequently, cells were incubated for 3 h at room temperature with primary antibodies diluted in antibody solution (5% normal goat serum, 0.1% Triton X-100, and 1% fatty acid-free BSA in PBS). Following three washes with PBS containing 0.1% Triton (PBST), cells were incubated for 1 h at room temperature in the dark with fluorescent-conjugated secondary antibodies. After additional PBST washes, coverslips were stained with DAPI (0.5 µg/ml, 15 min), washed, and mounted using Fluoroshield mounting medium (ImmunoBioScience, Mukilteo, WA). Slides were dried overnight at 4°C, and images were captured using a confocal microscope (Olympus IX81, Tokyo, Japan). Quantitation of protein levels, as reflected in the staining intensity, was performed using ImageJ software.

#### Ferroptosis assays

Ferroptosis was assessed in C6 cells treated with erastin and in HepG2 cells treated with acetaminophen (APAP), each for 24 h. Calcein‑AM, a cell‑permeable dye that becomes fluorescent upon cleavage by intracellular esterases. After treatment, cells were incubated with calcein‑AM (2 μM) for 30 min at 37 °C, washed with PBS, and imaged by fluorescence microscopy.

Ferroptosis in live cells was assessed using C11-BODIPY™581/591, a fluorescent probe-sensitive lipid peroxidation, as previously described [54].

Oxidation of polyunsaturated fatty acids induces a fluorescence emission shift in C11-BODIPY581/591™ from red (~ 590 nm) to green (~ 510 nm), enabling ratiometric analysis of lipid peroxidation via confocal microscopy. C6 cells (1 × 10^5^), seeded on polylysine-coated glass slides in 12-well plates, were treated with erastin for 24 h, while Hep-G2 cells were treated with APAP (24 h). Following treatment, cells were incubated with C11-BODIPY581/591™ (1 μM) for 30 min, washed with FBS-free growth medium (DMEM), and imaged using an Olympus IX81 confocal microscope (Tokyo, Japan). Image analysis was conducted with ImageJ software (National Institutes of Health), with at least six randomly selected fields analyzed per sample.

#### Pyroptosis and GSDME-induced pyroptosis assay

Cells were treated as described in figure legends and analyzed for GSDME-induced pyroptosis by immunoblotting with specific antibodies to detect the cleavage of GSDME, which resulted in the appearance of the N-terminus fragment. Pyroptosis and GSDME-induced pyroptosis in brain and colon sections were assessed by immunofluorescence staining for GSDMD and GSDME, respectively, using their corresponding antibodies.

#### VDAC1 overexpression and oligomerization analysis

Following treatment with the indicated reagents, cells were harvested and washed with PBS (pH 8.3). To assess VDAC1 oligomerization, cells were incubated with or without the cross-linking agent EGS (ethylene glycol bis(succiimidyl succinate), 100 µM) for 15 min at 30°C. Cell lysates were prepared, and protein concentrations were determined. For immunoblot analysis, 20 µg of total protein were used to assess VDAC1 expression levels, while 40–60 µg were loaded to evaluate VDAC1 oligomerization. Samples were resolved by SDS-PAGE and transferred to membranes for immunoblotting with anti-VDAC1 antibodies. Quantification of immunoreactive VDAC1 monomers, dimers, and higher-order oligomers was performed using ImageJ software.

#### Mitochondrial ROS production and intracellular Ca^2^⁺ measurement

Mitochondrial reactive oxygen species (mtROS) generation was assessed using MitoSOX™ Red (5 µM), a fluorogenic dye selective for mitochondrial superoxide in live cells, following the manufacturer’s protocol (Invitrogen, Grand Island, NY). Fluorescence intensity was measured by flow cytometry using an iCyt SY3200 system and analyzed with EC800 software (Sony Biotechnology, San Jose, CA).

Cytosolic calcium levels were measured using the calcium-sensitive fluorescent dye Fluo-4 AM. Following experimental treatment, cells (1 × 10⁶ cells/ml) were collected by centrifugation (1,500 × g, 5 min), washed with HBSS buffer (5.33 mM KCl, 0.44 mM KH₂PO₄, 138 mM NaCl, 4 mM NaHCO₃, 0.3 mM Na₂HPO₄, 5.6 mM glucose) supplemented with 1.8 mM CaCl₂ [HBSS( +)]. Cells were then incubated in the dark with 2 µM Fluo-4 AM in 200 µL HBSS( +) for 30 min at 37°C. Excess dye was removed by washing, and cells were re-suspended in 200 µL of fresh HBSS( +). Intracellular Ca^2^⁺ levels were immediately analyzed using an iCyt flow cytometer, collecting at least 10,000 events per sample. Fluorescence was detected in the FL1 channel, where Ca^2^⁺-bound Fluo-4 emits enhanced green fluorescence. Data analysis was performed using EC800 software.

#### Proximity ligation assay (PLA)

Proximity ligation assays (PLAs) were performed using the Duolink® In Situ Red Starter Kit (Sigma-Aldrich), following the manufacturer’s protocol, to assess protein–protein interactions between VDAC1 and HK-II, as well as with NLRP3 and ASC, as previously described [55].

Briefly, PC-3 and HeLa cells were seeded on 35-mm coverslips and cultured to approximately 60% confluency. Cells were pre-treated with VBIT-4 or VBIT-12 (10 μM) for 2 h, followed by exposure to cisplatin at the indicated concentrations for 48 h. After treatment, cells were washed with PBS, fixed in 10% formaldehyde (in PBS) for 15 min at room temperature, and permeabilized with 0.1% Triton X-100 in PBS for 15 min. Following a PBS wash, cells were blocked for 1 h at 37°C using Duolink® Blocking Solution.

For detection of protein proximity, cells were incubated with primary antibody pairs: mouse anti-NLRP3 (1:500) and rabbit anti-ASC (1:500) for NLRP3–ASC interaction, or mouse anti-VDAC1 (1:750) and rabbit anti-HK-II (1:500) for VDAC1–HK-II interaction. After 1 h incubation at room temperature, cells were treated with species-specific PLA probes (PLUS and MINUS), each conjugated to complementary oligonucleotides. Ligation and amplification were performed according to the kit instructions, and interaction signals were visualized as distinct red fluorescent dots using Texas Red-labeled detection reagents. Nuclei were counterstained with DAPI, and coverslips were mounted with Fluoroshield mounting medium. Confocal images were acquired using an Olympus IX81 microscope. Quantitative analysis of PLA signals (puncta per cell) was conducted using ImageJ software (NIH), based on fluorescence intensity across the entire cell area.

### Disease mouse models

All animal procedures were conducted in accordance with the ethical guidelines approved by the Institutional Animal Care and Use Committee (IACUC) of Ben-Gurion University.

#### Alzheimer’s disease (AD) mouse model and VBIT-4 treatment

The 5XFAD transgenic mouse model that co-expresses five AD-related mutations, which recapitulate key pathological features of familial AD, was used for in-vivo studies. Male 5XFAD mice were obtained from the Jackson Laboratory and crossed with female C57BL/6 RCC mice for colony maintenance.

VBIT-4 was initially dissolved in DMSO to prepare an 80 mg/ml stock solution, then diluted in drinking water to a final concentration of 0.0625 mg/ml. Based on an average daily intake of 8 mL per 25 g mouse, this corresponded to an approximate dosage of 20 mg/kg/day. Wild-type (WT) control mice (n = 10) were administered either vehicle (0.36% DMSO in drinking water) or VBIT-4 (n = 10) under an intermittent dosing regimen, twice weekly, with one day of water-only in between. 5XFAD transgenic mice were similarly divided into two groups: vehicle-treated (n = 8) and VBIT-4-treated (n = 9). At the end of the treatment period, mice were euthanized via intraperitoneal injection of a lethal dose of pentobarbital. Transcardial perfusion was performed through the ascending aorta with ice-cold PBS, followed by 4% paraformaldehyde (PFA) in PBS. Brains were extracted, post-fixed, and processed for downstream histological analyses.

#### Colitis mouse model and VBIT-4 treatment

A dextran sulfate sodium (DSS)-induced colitis model was used to evaluate the in-vivo effects of VBIT-12 with female C57BL/6 mice (6–7 weeks old, H-2b; Envigo, Israel). To induce colitis, mice were given 2% (w/v) DSS dissolved in drinking water for five consecutive days, followed by eight days of regular water, for a total study duration of 14 days. VBIT-12 treatment was initiated on day 3 by supplementing drinking water with the compound for the remainder of the study period. At the study endpoint, mice were euthanized by CO₂ asphyxiation. The entire large intestine, spanning from the ileocecal junction to the anus, was carefully removed, weighed, and measured length. A segment of the colon was snap-frozen in liquid nitrogen for molecular analysis, while the remaining tissue was fixed overnight in 4% formaldehyde in phosphate-buffered saline (PBS, pH 7.4). Samples were processed for histological immunoblotting evaluation.

#### Histological, immunohistochemistry (IHC), and immunofluorescence (IF) analyses of brain and colon tissues

Paraffin-embedded brain or colon sections (5-µm thick) were subjected for both IHC and IF staining. Antigen retrieval was performed by incubating the slides at 95–98 °C for 30 min in citrate buffer (10mM, pH 6.0). Sections were then washed with PBS containing 0.1% Triton X-100 (pH 7.4) to enhance membrane permeability. To block nonspecific binding, slides were incubated with 10% normal goat serum (NGS) for 2 h at room temperature, followed by overnight incubation at 4°C with primary antibodies (listed in Table 1).

For IHC, endogenous peroxidase activity was blocked by incubating the sections with 3% H₂O₂ for 15 min. Following wash with PBS containing 0.05% Tween-20 (PBST), sections were incubated for 2 h at room temperature with horseradish peroxidase (HRP)-conjugated secondary antibodies. HRP activity was envisioned using 3,3′-diaminobenzidine (DAB; ImmPact-DAB, Burlingame, CA), followed by hematoxylin counterstaining. Slides were mounted and scanned using a panoramic slide scanner signal intensity was quantified using HistoQuant software (3DHISTECH Ltd, Hungary).

For IF staining, fluorophore-conjugated secondary antibodies (see Table S1) were applied following primary antibody incubation. Nuclei were counterstained with DAPI (0.07 µg/mL), and sections were mounted with Fluoroshield mounting medium (ImmunoBioScience) and imaged using an Olympus IX81 confocal microscope with fixed acquisition parameters across all samples.

Quantitative analysis of fluorescence intensity was conducted using ImageJ software. For each tissue section, five randomly selected images were analyzed per section. Immunostaining was performed on brain or colon sections from at least three animals per group and repeated independently 2–3 times. Image analysis was conducted in a blinded manner by two independent authors.

#### TUNEL staining

Apoptotic cell death was assessed using a DeadEnd™ Fluorometric TUNEL Assay Kit (Promega, Madison, WI), following the manufacturer’s instructions. Paraffin-embedded brain or colon sections were first deparaffinized, rehydrated through a graded ethanol series, and equilibrated in PBS. Tissue permeabilization was performed using proteinase K (20 µg/ml in PBS), followed by post fixation in 4% paraformaldehyde for 15 min at room temperature. Slides were then incubated with terminal deoxynucleotidyl transferase (TdT) reaction mixture (1 h, 37 °C) in the dark to label DNA strand breaks. After rinsing in saline-sodium citrate buffer, sections were counterstained with PI (1 µg/mL) to visualize total nuclei.

After mounting with Vectashield anti-fade medium (Vector Laboratories, Burlingame, CA), the sections were imaged using an Olympus IX81 confocal microscope under standardized settings.

#### Statistical analysis

Data are expressed as mean ± SEM (n = 3 independent experiments). The level of significance of the differences between the control and treated samples was determined using an unpaired, *t*-test. Statistical significance is reported as *p* < 0.05 (*), p < 0.01 (**), p < 0.001 (***), and *p* < 0.0001 (****). The *p*-values in black and blue represent the significance of the indicated sample relative to the control and indicated compound-treated samples or mice, respectively.

## Results

### VBIT-4 and VBIT-12 mitigated cisplatin-induced apoptosis, VDAC1 oligomerization, ROS production, and intracellular [Ca^2+^]***i***

**Fig. 1:**
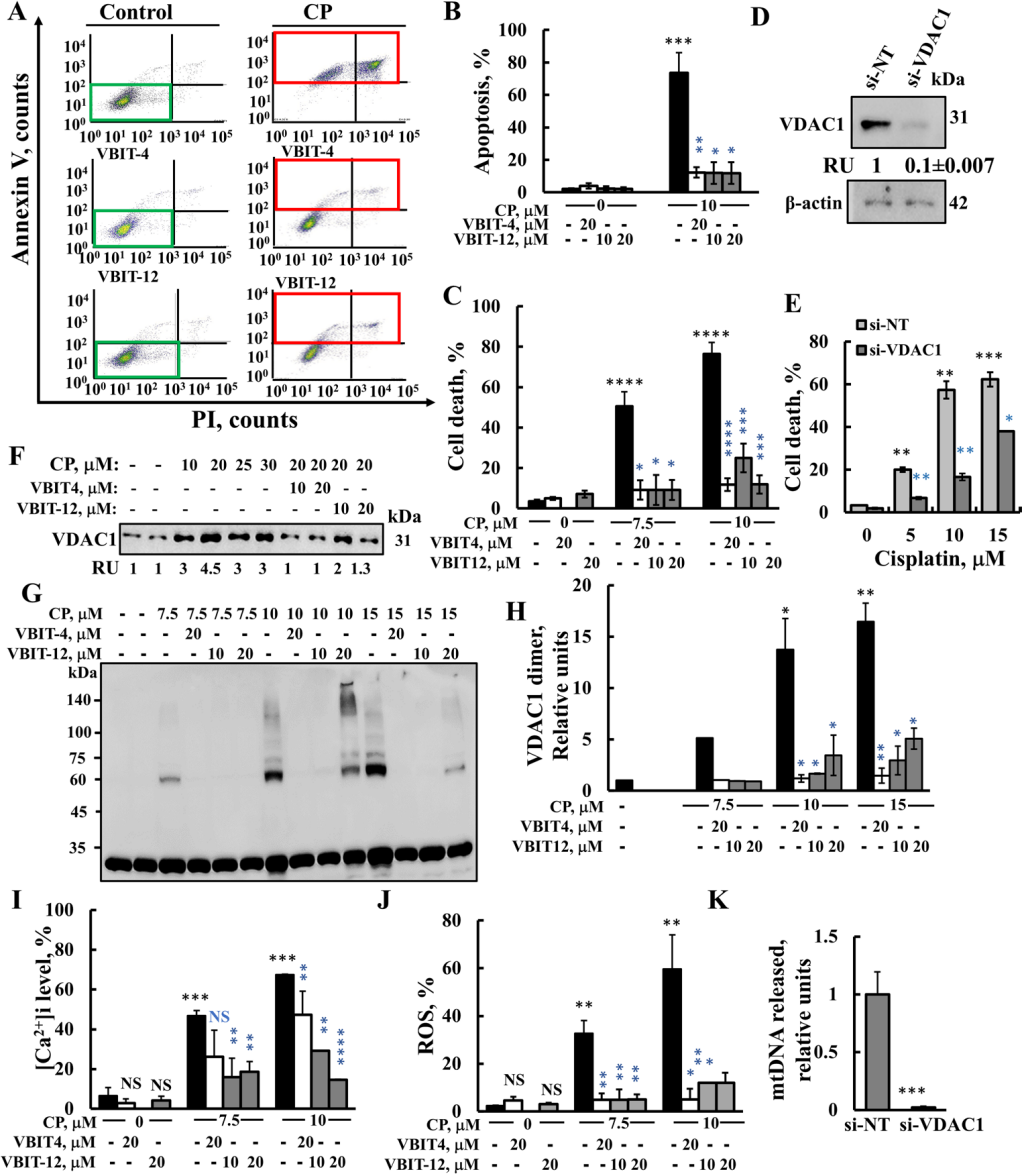
VBIT-4 and VBIT-12 protect against CP-induced apoptosis, VDAC1 oligomerization, and decrease intracellular [Ca^2+^]i and ROS levels and VDAC1 is required for mtDNA release. **A**, **B** HeLa cells were cultured in serum-free medium for 5 h, pretreated with the indicated concentrations of VBIT-4 or VBIT-12 for 2 h, and subsequently exposed to cisplatin (CP) for 48 h. Apoptosis was assessed by Annexin V-FITC/PI staining and analyzed by flow cytometry. Representative dot plots highlighting live (green box) and apoptotic (red box) cell populations are shown in (**A**), with quantification presented in (**B**). **C** Cell death was also evaluated by PI staining and flow cytometry under the same treatment conditions. **D**, **E** HeLa cells were transfected with 75 nM si-NT or with si-m/hVDAC1-B, using JetPrime transfection reagent, as described in the Methods section. At 24 h post transfection, cells were treated with the indicated concentrations of CP for 48 h and subjected to immunoblotting for VDAC1 expression levels using anti-VDAC1 specific antibodies and VDAC1 expression levels were quantified and are presented as relative units (RUs) below the blot (**D**). **E** Cell death induced by CP in these HeLa treated with si-NT or treated with si-m/h-VDAC1-B was determined using PI staining and a flow cytometer analysis. **F** Cells treated as described in (**A**) were collected, lysed, and analyzed by immunoblotting using anti-VDAC1 antibodies to assess total VDAC1 expression, and were quantified using ImageJ displayed as relative units (RUs) below the blot. **G**, **H** VDAC1 oligomerization was evaluated following chemical crosslinking with EGS (100 μM, 1 mg protein/ml) and immunoblotting (**G**), and quantification of oligomer levels was performed using ImageJ software (**H**). **I**, **J** Intracellular calcium levels ([Ca²⁺]ᵢ) were measured using Fluo-4-AM and flow cytometry (**I**), while mitochondrial ROS production was assessed with MitoSOX Red (**J**) following the same treatment protocol as in (**A**). **K** CT-26 cells were transfected with 50 nM of either si-NT or si-m/hVDAC1-B using JetPRIME. 48 h post transfection, cells were treated with 0.1 mM H₂O₂ for 20 h, and cytosolic mtDNA release was quantified as described in the Methods. Results are expressed in RUs, with si-NT–transfected cells set as 1.0. VDAC1 knockdown resulted in a substantial reduction in mtDNA release (<0.01 RU). Data are presented as the means ± SEM (n=3). Statistical significance: *p* ≤ 0.05 (*), *p* ≤ 0.01 (**), *p* ≤ 0.001 (***), *p* ≤ 0.0001 (****). Black and blue p-values indicate comparisons relative to the untreated control and CP-treated groups, respectively

Apoptosis, activated by various drugs, is associated with VDAC1 overexpression and oligomerization. This leads to pro-apoptotic factor release, caspases activation, and cell death induction [10, 18, 19, 39–42]. Here, we tested the effects of two inhibitors of VDAC1 oligomerization, VBIT-4 and VBIT-12, on apoptotic cell death and associated processes. HeLa cells incubated with cisplatin (CP) resulted in massive apoptosis (80%), as analyzed by annexin-V and PI, and flow cytometry (Fig. 1A, B) or PI staining (Fig. 1C). VBIT-4 and VBIT-12 strongly (90%) protected against CP-induced cell death (Figs. 1A–C, S1A, B).

The requirement of VDAC1 for CP-induced cell death was demonstrated by silencing VDAC1 using specific si-RNA and analyzing cell death (Fig. 1D, E). In HeLa cells with reduced VDAC1 expression, cisplatin-induced cell death was decreased by 50–80%, depending on the drug concentration. Moreover, the maximal level of cell death in VDAC1-deficient cells reached only ~ 50% of that observed in VDAC1-expressing cells (data not shown). Similar results were obtained with PC-3, A549 and HepG2, cell lines where the concentration of CP required to induce 50% cell death was 2.4–2.8 times higher when VDAC1 levels were reduced by 70–80% (Table S2).

These findings are consistent with previous studies demonstrating that VDAC1 plays a key role in CP-induced cell death. For example, silencing VDAC1, but not BAK, reduced CP-induced cell death [56]. Moreover, CP has been shown to bind directly to VDAC1, with the amount of CP associated with VDAC exceeding that bound to total cellular proteins by more than 200-fold [57]. In addition, exposure of cervical squamous carcinoma cells to CP led to upregulation of VDAC1, while in a CP-resistant subline (A431/Pt), VDAC1 expression was reduced threefold compared to the parental line [58].

As previously shown [19], CP-induced VDAC1 overexpression and oligomerization are linked to cell death (Fig. 1F-H). VBIT-4 and VBIT-12 inhibit not only cell death, but also VDAC1 oligomerization, as detected following chemical crosslinking and immunoblotting (Fig. 1F–H).

VBIT-4 and VBIT-12 effectively attenuated the CP-induced upregulation of VDAC1 expression (Fig. 1F). This reduction is not due to a direct effect of VBIT-4 or VBIT-12 on VDAC1 transcription or its post-translational modification, but rather, results from the prevention of CP-induced cell death or stress.

It was found that VDAC1 mRNA levels increased following CP treatment, suggesting that the observed increase in VDAC1 protein results from enhanced gene transcription. Furthermore, these compounds likely inhibit the activation of upstream signaling pathways and transcription factors that drive VDAC1 promoter activity [59].

Incubation with CP for 6, 12, 24, and 48 h revealed that VDAC1 levels were increase with the incubation time, reaching a maximal level at 48 h, whereas significant cell death was detected only after 48 h of treatment (Fig. S1C, D).

VBIT-4 and VBIT-12 also inhibited the CP- and selenite-induced Cyto *c* release (Fig. S1E,F) which is proposed to be mediated by the large channel formed within the VDAC1 oligomer [10, 18, 19, 39–42]. These results (Fig. S1C-F) suggest that the increase in VDAC1 levels represents an early event. Once VDAC1 reaches sufficiently high concentrations, it undergoes oligomerization, leading to Cyto *c* release and subsequent cell death.

The CP increased [Ca^2+^]*i*, as monitored using Fluo-4 and flow cytometry analysis, was prevented by VBIT-4 or VBIT-12 (Fig. 1I). These compounds also prevented CP-induced increase in the levels of mitochondrial ROS, as measured by MitoSOX Red (Fig. 1J).

As shown earlier [43, 44], VDAC1 oligomerization is required for mtDNA release. Silencing VDAC1 expression inhibited H₂O₂-induced mtDNA release (Fig. 1K), a process that triggers type-I interferon signaling and inflammation, as discussed below.

**Fig. 2:**
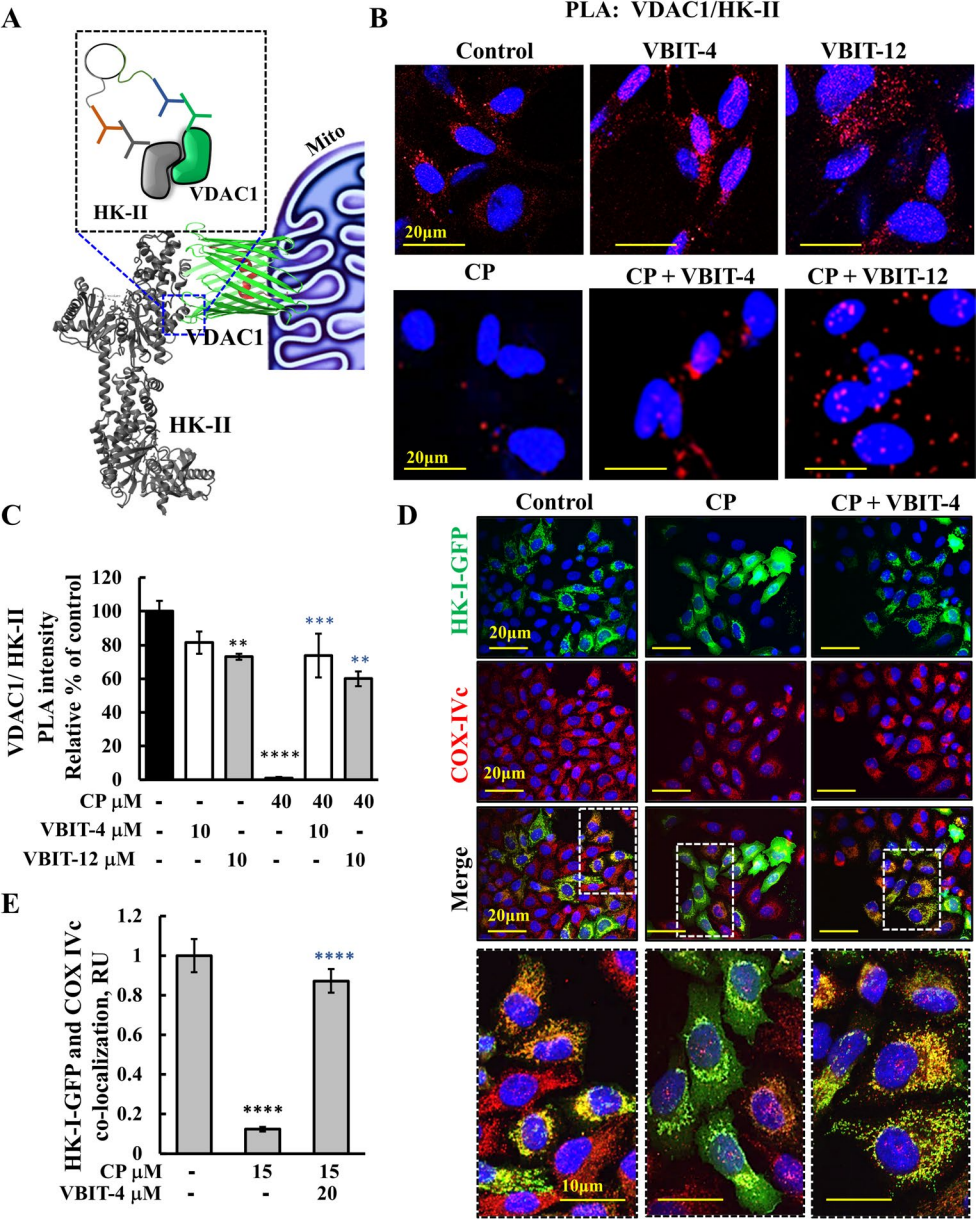
VBIT-4 and VBIT-12 prevent CP-induced disruption of HK-I and HK-II interactions with VDAC1, and detachment from mitochondria. **A** A schematic illustration of the interaction between HK-II and VDAC1 assessed by proximity ligation assay (PLA). **B, C** HeLa cells grown to ~ 60% confluence on 35-mm coverslips were pre-incubated with VBIT-4 or VBIT-12 (10 μM, 2 h) followed by CP treatment for 48 h at the indicated concentrations. Protein–protein interactions between HK-II and VDAC1 were detected via PLA using anti-HK-II (rabbit, 1:500) and anti-VDAC1 (mouse, 1:750) (**A**). Representative confocal images (**B**) and signal intensity quantification (**C**) are shown. **D** To visualize HK-I mitochondrial association, HeLa cells were transfected with pEGFP-HK-I (1μg) using JetPrime transfection reagent. After 24 h, cells were pre-incubated with or without VBIT-4 (20 μM; 2 h), followed by CP (15 μM; 48 h). Cells were then fixed and immunostained with anti–COX-VIc to label mitochondria and visualized for HK-I-GFP and COX-IVc using confocal microscopy. Representative images are shown from one of three independent experiments. **E** Quantification of co-localization of HK-I-GFP with COX-IVc was performed using ImageJ. Results represent the means ± SEM (n = 3), **p ≤ 0.01; ***p ≤ 0.001; ****p ≤ 0.0001. The *p*-values in black and blue represent the significance of the indicated sample compared to the control and CP-treated samples, respectively

The effect of VBIT-4 on mtDNA localization following CP treatment was also assessed using anti-dsDNA staining combined with co-immunostaining for mitochondrial markers Cyto c oxidase (COX) subunit IVc (COX-VIc) or VDAC1 (Fig. S2). In untreated control cells, dsDNA was predominantly co-localized with COX-VIc or VDAC1 (Fig. S2A-D), confirming its mitochondrial localization. However, upon CP treatment, dsDNA mitochondrial was primarily observed in the cytosol (Fig. 2A, B, E, F), with only limited co-localization with mitochondrial markers. Notably, treatment with VBIT-4 restored the mitochondrial localization of dsDNA in CP-treated cells. Similar patterns of mitochondrial and cytosolic mtDNA staining have been shown in mDPC6T cells following LPS-induced mitochondrial injury, as shown by confocal imaging [60] and in Acute retinal pigment epithelium (ARPE)-19 cells [61]. These results indicate that both VBIT-4 and VBIT-12 protect against cell death and associated mitochondria dysfunction processes.

### VBIT-4 and VBIT-12 prevented hexokinase detachment from VDAC1 induced by cisplatin

The mitochondria-bound isoforms HK-I and HK-II are highly expressed in cancer cells, increasing their efficiency in glucose usage [62], and they protect against cell death when attached to the mitochondria [53]. HK-I and HK-II are associated with the mitochondria via binding to VDAC1 [53, 62]. Here, the close association between VDAC1 and HK-II was analyzed using a proximity ligand assay (PLA) to detect protein–protein interactions that were < 40 nm in distance. By using specific anti-HK-II and anti-VDAC1 antibodies and imaging (Fig. 2A), we detected high PLA signals in control cells in the absence and the presence of VBIT-4 or VBIT-12. The signal was absent in the CP-treated cells, but its dissociation was protected by treatment with VBIT-4 or VBIT-12 (Fig. 2B,C). As expected, CP-induced cell death was prevented by VBIT-4 and VBIT-12 (Fig. S3A). The results indicate close proximity of the two proteins in control conditions with or without VBIT-4 or VBIT-12, which was not observed upon CP treatment. However, the proximity of HK-II and VDAC1 was restored by VBIT-4 and VBIT-12, thus, preventing HK-II detachment from the VDAC1, as previously found for HK-I [63].

To further demonstrate that VBIT-4 and VBIT-12 prevent CP-induced dissociation of HK-I from its mitochondrial binding site, cells expressing HK-I-GFP were used. Confocal imaging showed that HK-I-GFP cellular distribution in the control cells was punctate and co-localized with the electron transport chain protein COX-IVc, as expected in mitochondria binding (Fig. 2D). However, in cells exposed to CP, the HK-I-GFP fluorescence was diffused throughout the cytosol and did not co-localize with COX-IVc (Fig. 2D). Cell treatment with VBIT-4 or VBIT-12 prevented CP-induced HK-I-GFP detachment, as is also reflected in its co-localization with COX-IVc (Fig. 2E). These results indicate that the compounds prevented HK-I detachment from VDAC1, in agreement with previous studies [63].

### VBIT-4 and VBIT-12 inhibit ferroptosis, GSDME-induced pyroptosis/secondary necrosis, and NLRP3 inflammasome assembly

**Fig. 3:**
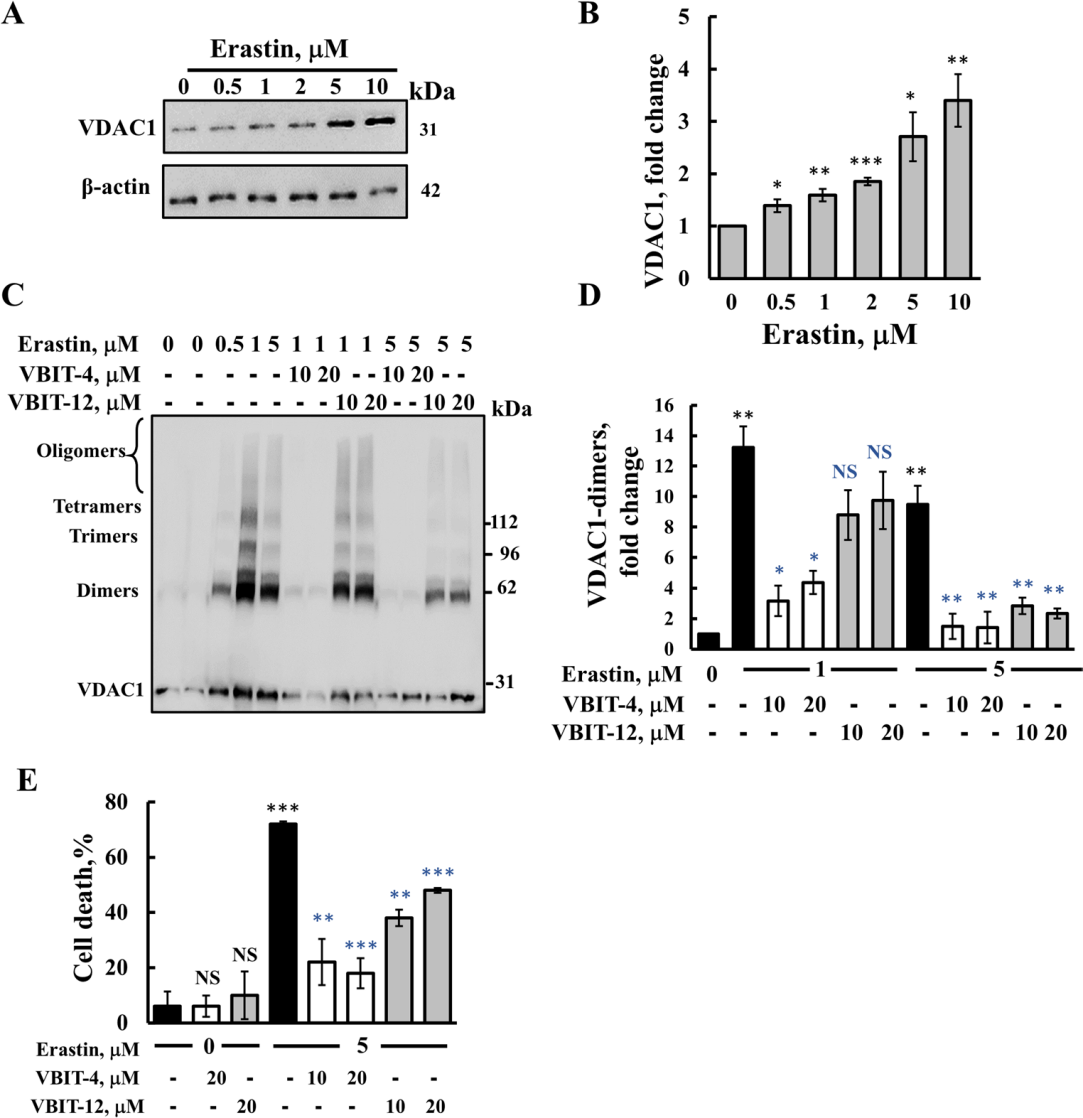
VBIT-4 and VBIT-12 suppress erastin-induced VDAC1 overexpression and oligomerization. **A, B** C6 cells were treated with or without the indicated concentrations of erastin (24 h), followed by lysis, and immunoblotting using anti-VDAC1 antibodies. β-actin served a loading control (**A**), and VDAC1 expression levels were quantified using ImageJ software (**B**). **C, D** To assess VDAC1 oligomerization, cells were cultured in serum-free medium for 5 h, pre-treated with VBIT-4 or VBIT-12 (10 or 20 μM, 2 h), and then incubated with erastin for 24 h. Cross-linking was performed using EGS (100 μM, 1 mg protein/ml), and VDAC1 oligomers were detected by immunoblotting (**C**). Densitometric analysis of VDAC1 dimers was performed using ImageJ (**D**). **E** Cell death under the same treatment conditions was evaluated by PI staining and analyzed by flow cytometry. Data are represented as the means ± SEM (n = 3). Statistical significance: *p ≤ 0.05; **p ≤ 0.01; ***p ≤ 0.001. Black and blue *p*-values indicate comparisons relative to untreated control and erastin-treated groups, respectively

**Fig. 4:**
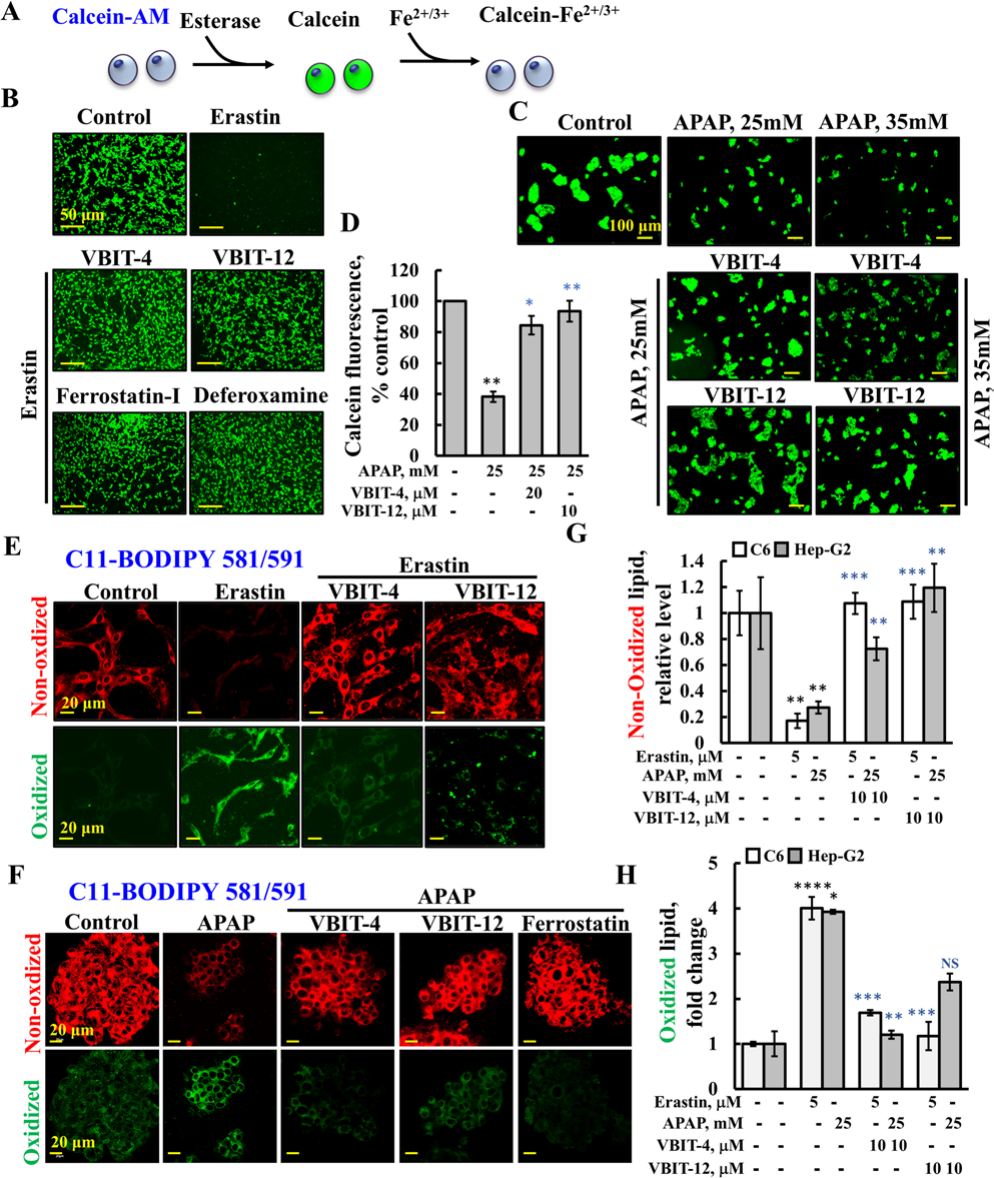
VBIT-4 and VBIT-12 suppress ferroptosis induced by erastin and acetaminophen (APAP). **A** Schematic reaction of calcein-AM monitoring ferroptosis. **B, C** C6 (B) and Hep-G2 (C) cells were serum-starved for 2 h, then pre-treated with VBIT-4 or VBIT-12 (10 μM or 20 μM; 2 h), followed by incubation with erastin (5 μM, 24 h; B;) or APAP (25 mM, 35 mM, 24 h; C). Cells were stained with calcein-AM to assess Fe^2^⁺/Fe^3^⁺ accumulation and imaged by confocal microscopy. **D** Quantification of calcein-AM fluorescence intensity from (B). **E, F** C6 (E) and Hep-G2 (F) cells were treated as described above, stained with C11-BODIPY581/591 to monitor lipid peroxidation, and live cells were imaged using confocal microscopy, with red and green forms of the probe indicating reduced and oxidized forms, respectively. Staining intensities of red (**G**) and green (**H**) fluorescence were analyzed using ImageJ software. Data represent the means ± SEM (n = 3). Statistical significance: *p ≤ 0.05; **p ≤ 0.01; ***p ≤ 0.001. Black and blue *p*-values indicate comparisons to untreated control and erastin- or APAP-treated groups, respectively

Ferroptosis is activated in C6 cells by erastin, which acts through the inhibition of system-Xc (SLC7A11), but it also interacts with VDAC2 [64]. As with CP in HeLa cells (Fig. 1A–H), erastin induced VDAC1 overexpression, oligomerization, and cell death, which were highly reduced in the presence of VBIT-4 or VBIT-12 (Fig. 3A–E). Ferroptosis was assayed by monitoring calcein-AM, which becomes fluorescent upon its cleavage by intracellular esterase, and quenched when it binds Fe^2+/3^ [65]. (Fig. 4A). Calcein-AM-incubated cells showed a high fluorescence signal that was highly reduced in the erastin-treated cells, and the fluorescence was not reduced in the presence of VBIT-4 and VBIT-12 (Fig. 4B). The highly reduced calcein signal represents the chelation of Fe^2+/3+^, and not of Ca^2+^, as the decrease in the calcein fluorescence signal induced by erastin was inhibited by the ferroptosis-specific inhibitors, ferrostatin-1 (which scavenges the alkoxyl radicals) [66] and deferoxamine (which chelates free iron) [67] (Fig. 4B). Ferroptosis was also analyzed by monitoring the expression levels of GPX4 which were highly reduced upon erastin treatment, but not in the presence of VBIT-4 (Fig. S4).

In Hep-G2 cells, ferroptosis is induced by APAP [68]. APAP exposure led to a marked reduction in calcein fluorescence, indicative of cell death; however, this decrease was effectively prevented in the presence of VBIT-4 and VBIT-12 (Fig. 4C, D).

The effects of VBIT-4 and VBIT-12 on lipid peroxidation, a hallmark of ferroptosis, were followed using C11-BODIPY. Confocal images of live cells show that in the control cells, C11-BODIPY was detected mainly as unoxidized lipids (red). The probe detected mainly peroxidized lipids (green) in the erastin-treated cells, while VBIT-4 or VBIT-12 treatment prevented erastin-induced lipid peroxidization (Fig. 4E, G, H). Similar results were obtained with Hep-G2 cells and APAP (Fig. 4 F–H).

Thus, in the different assays and cell lines, the results clearly show that ferroptosis induced by erastin or by APAP was inhibited by VBIT-4 and VBIT-12.

**Fig. 5:**
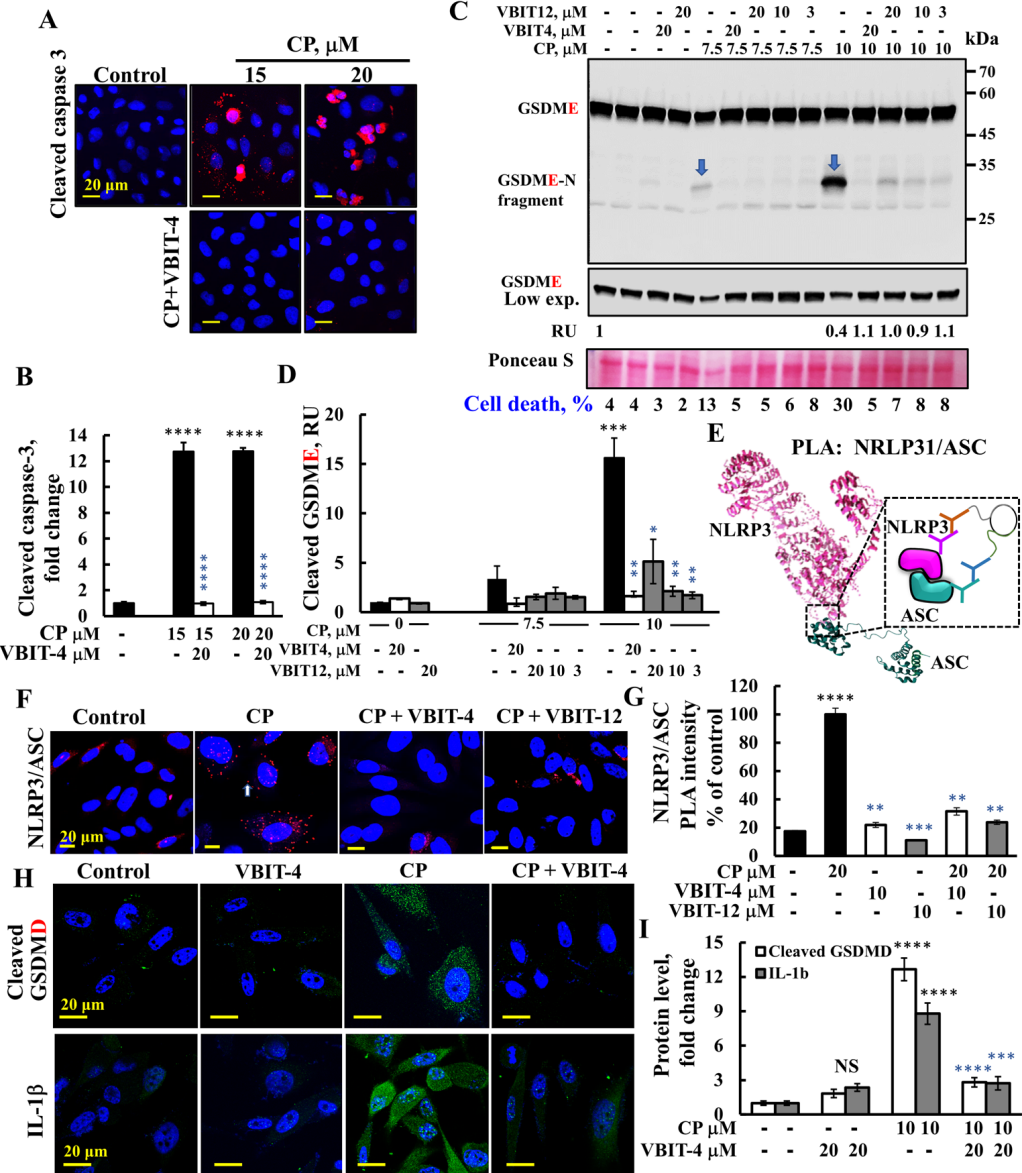
VBIT-4 or VBIT-12 protects against CP-triggered pyroptosis and secondary necrosis. **A, B** HeLa cells shown to express both NLRP3 and ASC [70], were cultured on 35-mm coverslips to ~ 60% confluence, pre-treated with VBIT-4 (20 μM, 2 h), and exposed to CP (15 or 20 μM, 48 h). Cells were fixed and stained with anti-cleaved caspase-3 antibody, followed by confocal microscopy (A). Quantification of cleaved caspase-3 fluorescence using ImageJ (B). **C, D** HeLa cells were pre-incubated with VBIT-4 or VBIT-12 (3, 10, or 20 μM, 2 h), then treated with CP (48 h). Cell lysates were analyzed by SDS-PAGE and immunoblotting with anti-GSDME antibodies (C), Ponceau S-stained blot as a loading control is shown. The levels of GSDME were quantified and showed as relative units (RUs) under the low exposure of the immunoblot. PI-based cell death analysis is also shown at the bottom. The N-fragment produced from cleaved GSDME was quantified (D). **E** Schematic presentation of NLRP3 and ASC interaction in a PLA assay. PC-3 cells were pre-incubated with VBIT-4 or VBIT-12 (10 μM, 2 h), then treated with CP (20μM, 24 h), and subjected to PLA using anti-NLRP3 (mouse, 1: 500) and anti-ASC (rabbit, 1:500), as described in the Methods section. Confocal images (F) and signal intensity quantification using ImageJ software (G) are shown. **H, I** PC-3 cells (2 × 10^5^ cells/well, 6-well plate) were pre-treated with VBIT-4 (20 μM, 2 h), and exposed to CP (10 μM, 42 h). Cells were fixed and stained with anti-cleaved GSDMD or anti-IL-1β antibody, followed by confocal microscopy and quantification using ImageJ. Data = means ± SEM (n = 3). Statistical significance: *p ≤ 0.05; **p ≤ 0.01; ***p ≤ 0.001; ****p ≤ 0.0001. Black and blue *p*-values indicate comparisons to untreated control and CP-treated samples, respectively.

We investigated the impact of VBIT-4 and VBIT-12 on pyroptosis, an inflammatory, caspase-dependent form of cell death initiated by intracellular sensors such as NLRP3 [20]. Pyroptosis is characterized by the activation of caspase-1, which cleaves GSDMD producing an N-terminal fragment that forms pores in the plasma membrane, leading to membrane permeabilization and the release of pro-inflammatory mediators [6, 7]. GSDME-mediated pyroptosis/secondary necrosis, driven by caspase-3 activation, is shown in Fig. 5.

Treatment with CP led to robust caspase-3 activation in HeLa cells, as evidenced by elevated levels of cleaved caspase-3, which was significantly attenuated by VBIT-4 (Fig. 5A, B). Caspase-3-mediated cleavage of GSDME, resulting in the pore-forming GSDME-N fragment, was also prevented by VBIT-4 and VBIT-12 (Fig. 5C, D). This GSDME-induced pyroptosis is also referred to as post-apoptosis or secondary necrosis.

To further examine the involvement of canonical inflammasome signaling, we assessed NLRP3 inflammasome activation following CP treatment with and without VBIT-4 and VBIT-12. NLRP3 is composed of members of the NOD-like receptor family, initiating inflammasome assembly through interaction with the adaptor protein ASC (apoptosis-associated speck-like protein), which recruits and activates caspase-1. A PLA using anti-NLRP3 and anti-ASC-specific antibodies (Fig. 5E) demonstrated a marked increase in the PLA signal upon CP exposure, indicating NLRP3 inflammasome activation. This increase was abolished in the presence of either VBIT-4 or VBIT-12 (Fig. 5F, G). CP-induced cell death was significantly reduced in cells pre-treated with VBIT-4 or VBIT-12 (Fig. S3B). These findings align with prior reports implicating VDAC1 oligomerization as a critical step in NLRP3 inflammasome assembly [45].

As expected [69], upon CP-induced of NLRP3 inflammasome activation and subsequent activation of caspase-1, led to a high increase in cytokines IL-1β (ninefold) and cleaved GSDMD (12-fold) levels, which was reduced by VBIT-4 in PC-3 cells that express GSDMD (Fig. 5H,I).

### VBIT-4 and VBIT-12 inhibit PCD, apoptosis, pyroptosis, and ferroptosis in disease mouse models

Different types of PCD are activated in several diseases [4–12, 20]. In the current study, we selected ***Alzheimer’s disease*** (AD) and ***inflammatory bowel diseases*** (IBD) to test the activation of apoptosis, pyroptosis, and ferroptosis, and their inhibition by VBIT-4 or VBIT-12. Both compounds were evaluated in the two disease models [46, 47]. In this study, we present results obtained with VBIT-4 in the AD model and VBIT-12 in the IBD model, as each compound exhibited the strongest effect in its respective disease model.

**Fig. 6:**
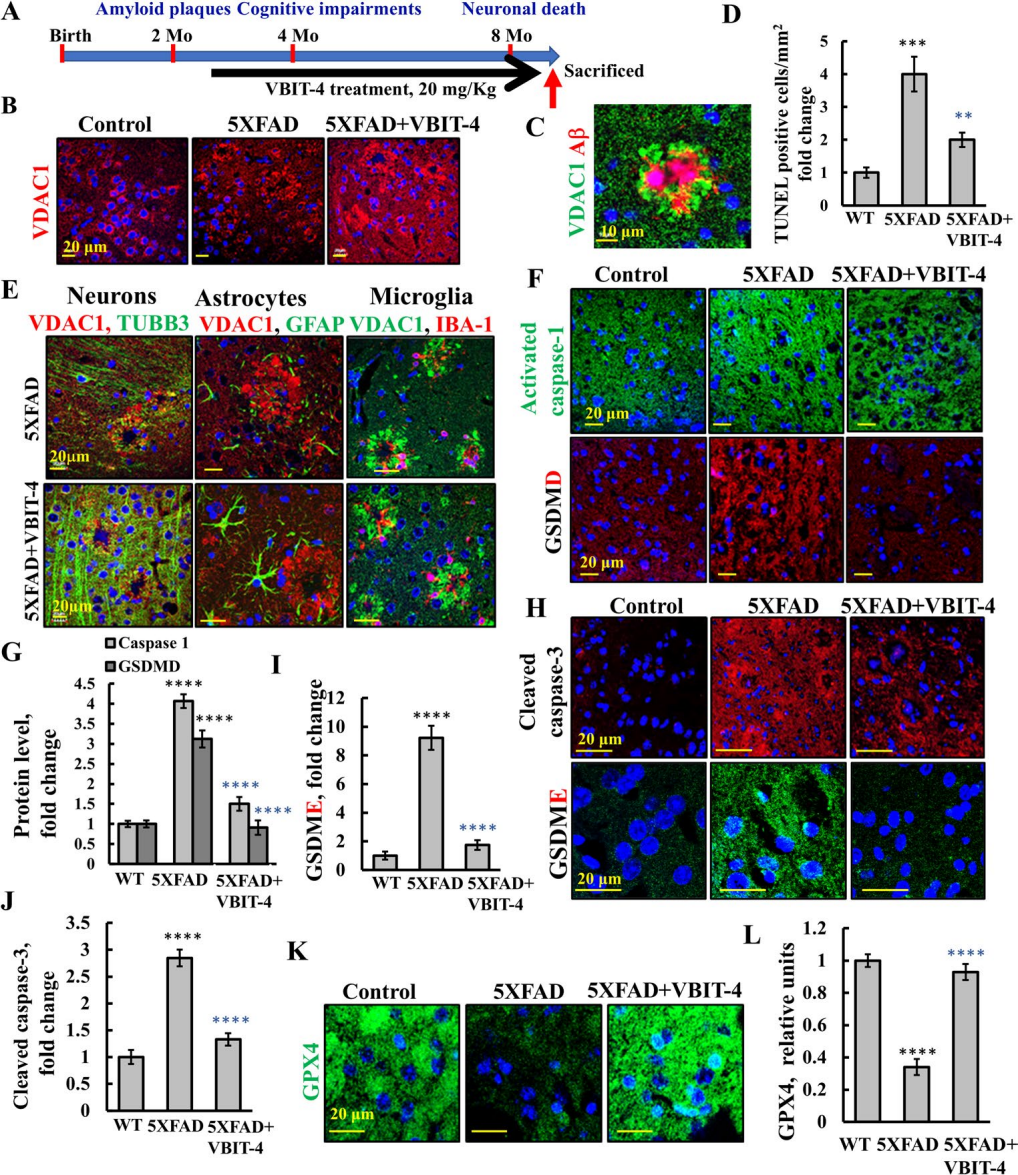
VBIT-4 alleviates neuropathology in 5XFAD mice by inhibiting apoptosis, pyroptosis, and ferroptosis**. A** Schematic representation of the experimental timeline outlining disease progression in 5XFAD mice and the VBIT-4 treatment regimen. **B, C** Representative IF images of cortical sections from WT and 5XFAD mice, with or without VBIT-4 treatment, stained for VDAC1 expression (B), or co-IF stained with anti-Aβ (red) and anti-VDAC1 (green) (C) antibodies, reveal VDAC1 overexpression in ring-like structures surrounding Aβ plaques. **D** Quantification of apoptotic cells in cortical sections by TUNEL assay, presented as the average number of TUNEL-positive cells per mm^2^ and normalized as fold change relative to WT controls. **E** Representative confocal images of cortical sections from untreated and VBIT-4-treated 5XFAD mice co-immunostained with antibodies against VDAC1 and cell-specific markers: TUBB3 for neurons, GFAP for astrocytes, and IBA-1 for microglia. Nuclei were counterstained with DAPI. **F, G** IF staining of cortical sections for pyroptosis markers: cleaved GSDMD and active caspase-1, and their quantification (G). **H–J** IF analysis of cleaved GSDME and activated caspase-3 with corresponding quantification of signal intensities in (I) and (J), respectively. **K, L** Confocal images of GPX4 expression in cortical sections (K), and quantification of its levels (L). Data are presented as the means ± SEM (n = 3 mice), **p < 0.01, ***p < 0.001, ****p < 0.0001. Black and blue asterisks denote significance relative to WT and untreated 5XFAD groups, respectively

Previously, using the AD-like mouse model 5XFAD (Fig. 6A), we showed that VDAC1 is highly overexpressed in ring-like patterns in brain cortex sections (Fig. 6B), corresponding to neutrophils surrounding Aβ plaques (Fig. 6C). VDAC1 levels in the cells around the Aβ − plaques were approximately 15-fold higher than in the surrounding tissue (Table S3). The brains of 5XFAD mice exhibited neuronal loss, neuroinflammation, apoptotic cell death, and morphological changes in astrocytes and microglia—all of which were prevented by VBIT-4 treatment [46]. Overall cell death, assessed by TUNEL staining (Fig. 6D), and neuronal loss, indicated by reduced expression of the neuronal marker TUBB3 (Fig. 6E, Table S3), were significantly prevented in the VBIT-4-treated 5XFAD mice.

VBIT-4 also prevented astrocyte and microglia morphological alteration. Immunofluorescent staining of the astrocytes in the cortical brain tissue, using the cytoskeletal marker GFAP, revealed improved cellular morphology in the VBIT-4–treated 5XFAD mice. These astrocytes exhibited increased branching, longer processes, and an expanded surface area, indicating improved structural integrity and activation state (Fig. 6E).

Microglia, key mediators of neuroinflammation and Aβ clearance, undergo pronounced morphological changes upon activation, typically adopting a rounded shape with retracted processes [71]. In the untreated 5XFAD mice, IBA-1 staining revealed microglia with hypertrophic cell bodies and short, thick processes—hallmarks of a pro-inflammatory phenotype. In contrast, the microglia from the VBIT-4-treated mice displayed a more ramified morphology, characterized by longer and more numerous processes (Fig. 6E). These observations indicate that VBIT-4 exerts a protective effect on glial cells, preserving both astrocytic architecture and microglial functionality. This aligns with prior findings showing that VBIT-4 enhances glial metabolic activity and contributes to overall neuroprotection [46].

The protective effects of VBIT-4 against AD-related pathogenesis prevent deterioration of hippocampal-dependent learning and memory [46]. The activation of cortical pyroptosis in 5XFAD mice and the effect of VBIT-4 were quantified by IF staining of GSDMD and caspase-1 which showed increased staining in the 5XFAD mice that was highly reduced in the VBIT-4-treated mice (Fig. 6F,G). This agrees with our previous findings of inflammation activation in the 5XFAD brain that is prevented by VBIT-4 [46].

GSDME is cleaved by activated caspase-3, whose levels were increased in the 5XFAD group, leading to GSDME-induced pyroptosis, but not in the group treated with VBIT-4 (Fig. 6H–J).

In addition, ferroptosis activation and the impact of VBIT-4 were assessed in cortical sections from WT and 5XFAD mice using IF staining with anti-GPX4 antibodies. GPX4 levels were reduced in 5XFAD mice, whereas VBIT-4 treatment preserved GPX4 expression (Fig. 6K, L). These results indicate that in the brains of 5XFAD mice, in addition to apoptosis, ferroptosis, both pyroptosis and GSDME-induced pyroptosis were also activated in AD-like mice and highly reduced in the VBIT-4-treated mice.

**Fig. 7:**
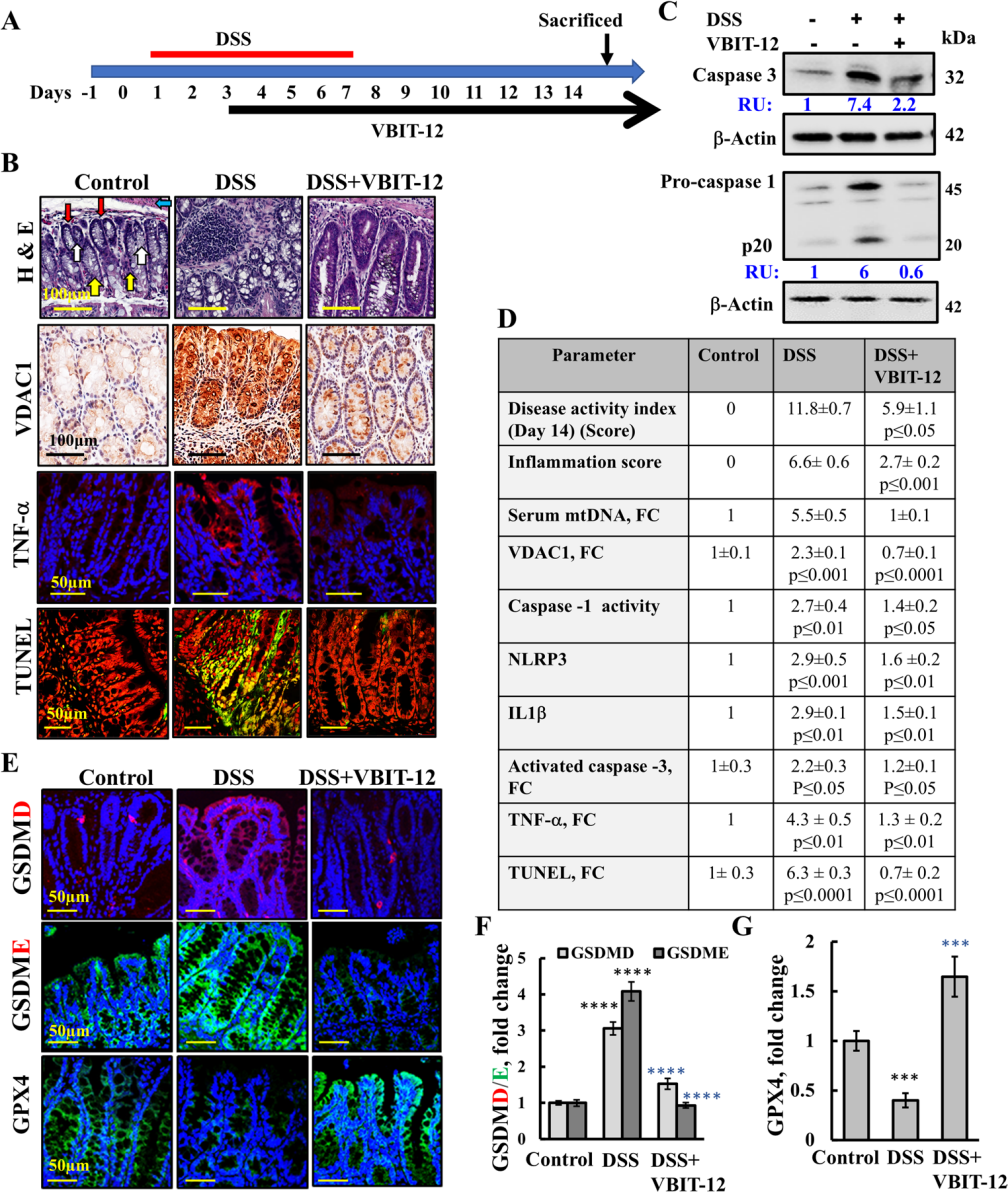
VBIT-12 alleviates pathological features of DSS-induced colitis by inhibiting apoptosis, pyroptosis, and ferroptosis. **A** Experimental timeline illustrating the induction of colitis via 2% DSS administered in drinking water for 5 days, followed by 8 days of regular water. VBIT-12 treatment (20 mg/kg) was initiated on day 2 and continued throughout the study (see Methods). Mice were sacrificed on day 14. **B** Representative hematoxylin and eosin- (H&E)-stained colon sections from control, DSS-treated, and DSS + VBIT-12-treated mice. Red, yellow, blue, and white arrows indicate crypt structures, lamina propria, submucosa, and lumen, respectively. Sections were also IHC-stained for VDAC1 and IF-stained for TNF–α or subjected to TUNEL staining, where cells stained green/yellow were identified as apoptotic. **C** Protein extracts from colon tissues were analyzed by immunoblotting for cleaved caspase-3, and pro-caspase-1 and its 20kDa cleavage product, with β-actin as a loading control. Densitometric quantification is shown in relative units (RUs). **D** The table summarizes the disease activity index, inflammatory score, serum mtDNA, expression level of VDAC1, TNF-α, activated caspase-3, caspase-1 activity, NLRP3 and IL-1β (immunoblotting), and cell death (TUNEL). FC–fold change relative to control. **E–G** Representative immunofluorescence images of colon sections stained for pyroptotic markers GSDMD and GSDME, and the ferroptosis marker GPX4, comparing untreated, DSS-treated, and DSS + VBIT-12-treated groups (E), and quantification of their fluorescence intensity (F,G). Data are expressed as the means ± SEM (n = 3 mice), ***p < 0.001, ****p < 0.0001. Black and blue *p*-values represent the significance of the indicated sample compared to the control and DSS-treated mice, respectively

Next, we evaluated the effects of VBIT-12 on colitis, as induced in the mouse colon using DSS [47] (Fig. 7A). VBIT-12 protected against the pathology of DSS- or trinitrobenzene sulphonic acid (TNBS)-induced colitis [47]. In these mice, VBIT-12 treatment prevented the epithelial cell damage and focal inflammation observed in the colons of DSS-treated mice (Fig. 7B). VDAC1 levels, cell death (TUNEL staining and activated caspase-3), inflammation (TNF-α, and inflammation score), and serum mtDNA were all highly increased in the DSS-treated mice, but not in those treated with VBIT-12 (Fig. 7B, D).

The induction of ferroptosis and pyroptosis in the colon of the DSS-treated mice and the effect of VBIT-12 on these forms of PCD types were evaluated (Fig.  7C, E–G). Caspase-1, upon recruitment to inflammasomes, mediates the proteolytic maturation of the cytokines IL-1β and IL-18 [69]. The DSS-treated mice showed elevated levels of pro-caspase-1 and its cleaved p20 subunit, both of which were reduced in the mice treated with VBIT-12 (Fig. 7C). Additionally, DSS treatment led to increased caspase-1 activity, serum mtDNA, NLRP3, and IL-1β levels—all of which were inhibited by VBIT-12 (Fig. 7C, D).

These results support the activation of pyroptosis. Further evidence is provided by immunofluorescence staining of GSDMD in colon sections, which showed increased expression in the DSS-treated mice—an effect not seen with VBIT-12 treatment (Fig. 7E, F).

As shown in Fig. 7C, D, caspase-3 was activated in the DSS-treated mice. In addition to inducing apoptosis, caspase-3 also cleaves GSDME whose levels were elevated more than fourfold in the DSS-treated mice but returned to near-normal levels following VBIT-12 treatment (Fig. 7E, F). These findings suggest that apoptosis, mediated by caspase-3, progresses to secondary necrosis in this model, and that VBIT-12 effectively prevents this process.

Ferroptosis was revealed by colon sections IF-stained with anti-GPX4 antibodies, showing a decrease (70%) in GPX4 levels in the DSS-treated mice that was not observed in the VBIT-12-treated animals (Fig. 7E, G). These results indicate that in DSS-treated mice, in addition to apoptosis, ferroptosis, pyroptosis and GADME-induced pyroposis/secondary necrosis were also activated in the colon and were highly reduced in the VBIT-12-treated mice.

## Discussion

**Fig. 8:**
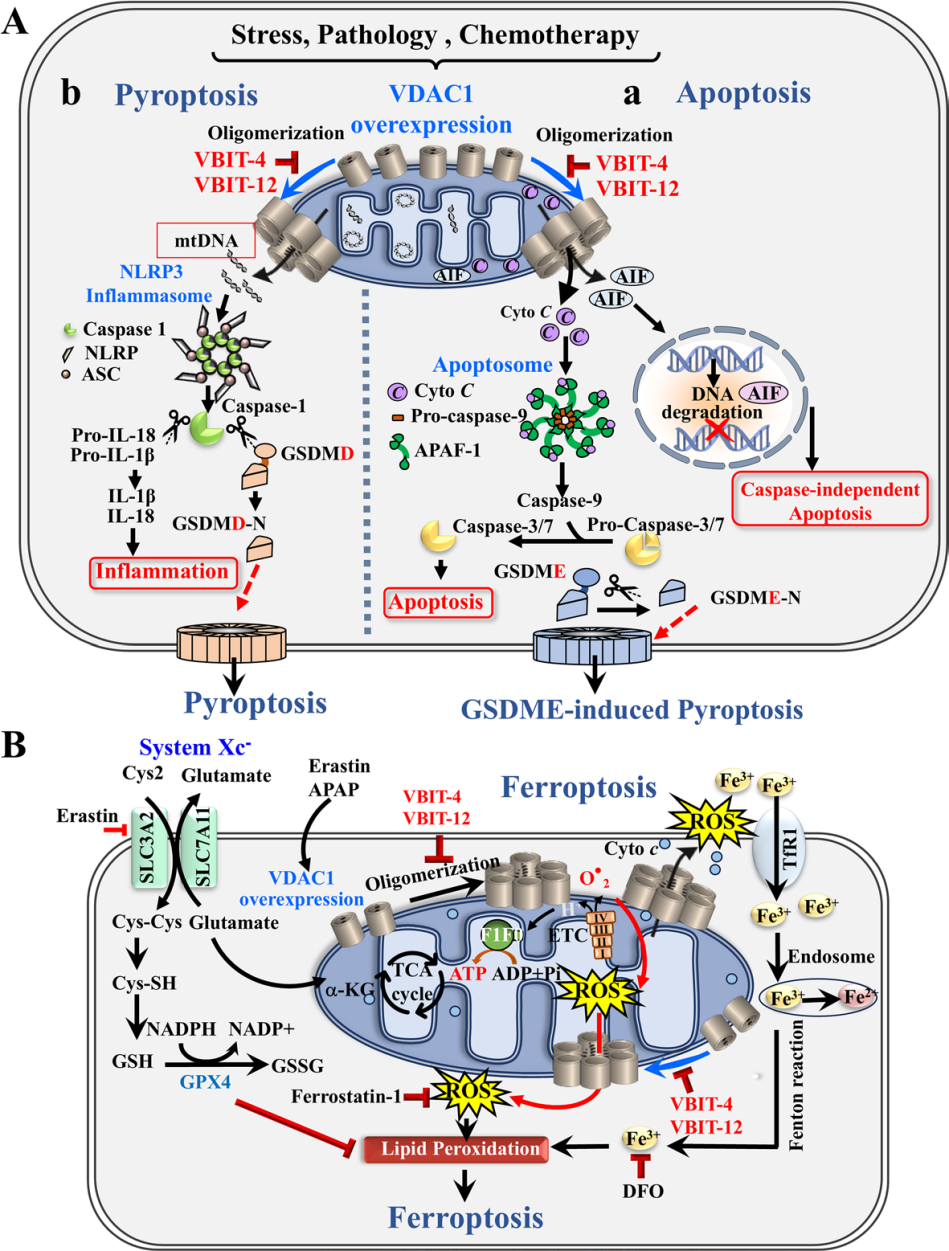
Schematic illustration of the pathways leading to apoptosis, pyroptosis, and ferroptosis via VDAC1 oligomerization, and their inhibition by VBIT-4 and VBIT-12. **A** Mitochondria-mediated apoptosis and pyroptosis, and their inhibition by VBIT-4 and VBIT-12: Intrinsic apoptosis is initiated by various cellular stressors—such as UV irradiation, DNA damage, chemotherapeutic agents, ROS, and Ca^2+^ overload, leading to overexpression and oligomerization of VDAC1 at the OMM. These large channels formed: (**a**) (Cyto c, AIF) released from the IMS into the cytosol. Cyto *c* forms a complex with Apaf-1 and procaspase-9 to assemble the apoptosome, which activates caspase-9, subsequently, activating executioner caspases-3/7, leading to apoptosis. AIF is cleaved by calpains or cathepsins through VDAC1 oligomer pores and translocates to the nucleus inducing DNA fragmentation, causing caspase-independent apoptosis. Inhibiting VDAC1 oligomerization blocks both caspase-dependent and -independent apoptosis. Apoptosome-activated caspase-3 also cleaves GSDME, resulting in its N-terminal fragments which form pores in the plasma membrane, initiating secondary necrosis or GSDME-dependent pyroptosis. (**b)** VDAC1 oligomerization facilitates the release of mtDNA, which contributes to inflammasome activation (e.g., NLRP3) and caspase-1. Caspase-1 cleaves GSDMD, releasing their N-terminal fragments that form membrane pores, triggering pyroptosis. VBIT-4 and VBIT-12, by blocking VDAC1 oligomerization, protect against both apoptosis and pyroptosis. **B** VDAC1 oligomerization in ferroptosis and its inhibition by VBIT-4/12: VDAC1-oligomers mediate ferroptosis and protection by VBIT-4/12. Schematic presentation of the proposed erastin and APAP mode of action in ferroptosis induction and the protection by VBIT-4 and VBIT-12. System Xc⁻ antiporter imports cystine in exchange for glutamate. Cystine is converted to cysteine, producing GSH, a key cofactor for GPX4, preventing lipid peroxidation. Erastin inhibits this transporter, depleting GSH and increasing ROS, leading to lipid peroxidation. Fe^3+^, imported through the transferrin receptor, is reduced to Fe^2+^ in the endosomes and reoxidized through Fenton chemistry, enhancing lipid peroxidation via lipoxygenases. Both erastin (Fig. 3A–D) and APAP [49] induce VDAC1 overexpression and oligomerization, resulting in Cyto *c* release and ROS production that escapes through VDAC1 oligomers, elevating cytosolic ROS, and promoting ferroptosis. VBIT-4 and VBIT-12 prevent VDAC1 oligomerization, thereby protecting against erastin- and APAP-induced ferroptosis

In this study, through a series of cellular, molecular, and disease mouse models, we demonstrated that mitochondrial VDAC1 plays a role not only in apoptosis, but also in pyroptosis, GSDME-induced pyroptosis (or secondary necrosis), and ferroptosis. These different forms of PCD can coexist within individual cells or tissues, and they share common molecular players that can be simultaneously activated, thus, leading to complex interactions among them (Fig. 8) [1–3]. This plasticity among the PCD pathways means that blocking one form of PCD may trigger the activation of other forms. Therefore, developing a global inhibitor that targets all forms of PCD is crucial, and particularly applicable for exploring therapeutic interventions.

Currently, there are no known inhibitors capable of simultaneously blocking all types of PCD. However, our findings demonstrate that the mitochondria and VDAC1 are involved in all tested PCD pathways and can be effectively inhibited by a single VDAC1-interacting molecule—either VBIT-4 or VBIT-12. These compounds represent the first global inhibitors capable of blocking the several tested forms of PCD.

### VBIT-4 and VBIT-12 inhibit multiple forms of PCD

Mitochondrial dysfunction contributes to diseases by impairing energy and metabolite production, increasing ROS, and triggering mitochondrial-mediated PCD, including apoptosis, secondary necrosis, pyroptosis, and ferroptosis. It also triggers the release of mtDNA, which activates the cGAS-STING pathway and promotes inflammation. [43, 72]. VDAC1 overexpression and oligomerization are central to these processes, linking mitochondrial dysfunction to apoptosis, pyroptosis, ferroptosis, and inflammation [4–10, 12, 37, 38, 53, 54] (Figs. 1, 2, 3, 4, 5, 6 and 7) and to diseases (Table 1).

**Table 1:** Diseases associated with VDAC1 overexpression—VBIT-4 or VBIT-12 protects against the pathological conditions

Disease	VDAC1 expression	Effect on VDAC1 oligomerization and/or expression	Effects of VBIT-4 or VBIT-12	Refs
CNS (neurodegenerative diseases)				
1. Alzheimer’s disease	Overexpressed	In vitro: VBIT-4 decreased VDAC1 oligomerization and expression. In vivo: VBIT-4 reduced VDAC1 expression	VBIT-4/VBIT-12 protected again neuronal cell death and inflammation, restored astroglia and microglia morphology, and improved cognitive activity in 5XFAD mice. VBIT-4 improved cognitive function in APP/PS1 mice	[46, 73]
2. Parkinson’s disease	Overexpressed	Not tested	VBIT-12 prevented the loss of dopaminergic neurons	unpublished
3. Retinal neuron injury	Not tested	VBIT-12 inhibited VDAC1 oligomerization	VBIT-12 inhibited VDAC1 oligomerization, apoptosis, and necroptosis, and improved mitochondrial function in retinal neurons	[74]
4. VBIT-12 rescued Age related Macular Degeneration (AMD)	Not tested	VBIT-12 inhibited VDAC1 oligomerization and leakage of mtDNA	VDAC1 oligomerization promotes mtDNA release and activates the cGAS–STING signaling pathway in age-related macular degeneration (AMD). Treatment with VBIT-12 significantly reduces mtDNA efflux, inhibits STING–PANoptosome complex formation, and preserves retinal pigment epithelial (RPE) cell function	[61]
5. Sleep deprivation and chronic pain	Overexpressed, also in plasma membrane	VBIT-4 reduced both monomeric and oligomeric VDAC levels in vitro and in vivo	VBIT-4 restored LPS-impaired microglial function by reducing ATP release and IL-1β and CCL2 expression. It also reversed microglial activation and prevented persistent pain after sleep deprivation in mice	[75]
6. Amyotrophic lateral sclerosis (ALS)	Overexpressed	VBIT-4 reduced VDAC1 monomers, dimers and oligomers, ex vivo	VBIT-12/VBIT-4 rescued cell death induced by mutant SOD1 in neuronal cultures, and improved muscle endurance in mutant SOD1G93A mice	[76]
7. Epilepsy	Overexpressed in the epileptogenic zone of a drug-resistant epileptic model	Not tested	VBIT-4 reduced seizure frequency and behavioral disturbances in epileptic mice maintained on a ketogenic diet, while also attenuating neuroinflammation and oxidative stress	[77, 78]

Metabolic diseases				
8. Hyperglycemia	Increased mRNA levels in lung endotheliocytes and decreased in skin fibroblasts	Not tested	VBIT-4 abolished the hyperglycemia-induced increase in MPT pore and ROS generation	[79]
Hyperglycemia	Overexpressed, also in plasma membrane	VDAC1 expression reduced in vitro	VBIT-4 prevented retinopathy and protected against cytokine-reduced cell viability	[80]
9. Type 2 diabetes beta cell dysfunction	Overexpresse, also in plasma membrane	Not tested	VBIT prevented hyperglycemia by preserving ATP levels, enhancing insulin secretion, maintaining glucose homeostasis, and improving glucose-stimulated insulin secretion	[48, 81]

Inflammatory and immune diseases				
10. Systemic lupus erythematosus	Overexpressed	VBIT-4 decreased VDAC1 oligomerization in vivo	VBIT-4 inhibited mtDNA release and type-Ι interferon responses, and lupus pathologies	[43]
11. Viral immunity	Not tested	Not tested	VBIT-4 inhibited mtDNA release to cytosol, triggering c-GAS, STING, and INF-I signaling, and an anti-viral response in CLPP-KO cells	[82]
12. Colitis, Crohn’s disease	Overexpressed	VBIT-12 decreased VDAC1 oligomerization in vitro	VBIT-12 inhibited apoptosis, inflammation, and an immune response	[47]
13. Periodontitis		Not tested	VBIT-4 inhibited mtDNA-induced inflammation	[83]
14. Inflammation	Overexpressed	VBIT-4 decreased VDAC1 oligomerization in vitro	VBIT-4 inhibited VDAC1 oligomerization, mtDNA release, and inflammasome assembly and activation	[45, 72]
15. COVID-19	Overexpressed	Not tasted	VBIT-4 suppressed activation of the cGAS-STING signaling pathway during SARS-CoV-2 infection	[51, 52]

Toxicity, ischemia, and other diseases				
16. Acute liver injury	Overexpressed	Not tasted	VBIT-12 inhibited APAP-induced hepatocyte cell death in vitro via ferroptosis	[49]
17. Renal ischemia –reperfusion injury (AKI)	Overexpressed	Not tasted	VBIT-12 reduced creatinine levels and prevented apoptosis	unpublished
18. Kidney fibrosis	Overexpressed	Not tasted	VBIT-12 attenuated renal injury and inflammation	[84]
19. Myocardial injury	Overexpressed	Not tested	VBIT-4 inhibited cardiomyocyte cell death and fibrosis	[85]
20. Alcoholic liver disease (ALD)	Oligomerized	Not tasted	VBIT-12 preserved mitochondrial function	[86]
21. Pigmentary disorders	Oligomerized	Not tasted	VBIT-12 promoted melanogenesis and pigmentation in epidermal melanocytes in zebrafish and human skin explants	[87]
22. Duchenne Muscular Dystrophy (DMD)	Not tasted	Not tasted	In a severe model of DMD, VBIT-4 rescues mitochondrial dysfunction, including reduced mitochondrial Ca^2+^ overload, decreased PTP induction, improved mitochondrial ultrastructure and reduces skeletal muscle degeneration	[88]
23. Myofiber senescence in Zmpste24⁻/⁻ (Zinc metallo-peptidase STE24) mice, a model of Hutchinson-Gilford Progeria Syndrome		VBIT-4 inhibition of VDAC1 oligomerization in Z24 − / − myofibers	In myofibers from Zmpste24⁻/⁻ mice, increased mtDNA release was associated with enhanced VDAC1 oligomerization. Treatment with VBIT-4, reduced mtDNA release and downstream cGAS-STING pathway activation	[89]

The various diseases are sub-grouped, and the expression levels of VDAC1, along with the effects of VBIT-4 or VBIT-12 treatment on the disease pathology are indicated, as well as the related publication.

As depicted in Fig. 8A, overexpression of mitochondrial VDAC1 promotes its oligomerization, forming large pores that facilitate the release of key pro-apoptotic and pro-inflammatory factors, thereby initiating apoptosis and inflammation, rely. In response to apoptotic stimuli, Cyto *c* is released through these VDAC1 oligomers into the cytosol, where it promotes apoptosome assembly and the subsequent activation of caspase-9 and caspase-3, ultimately driving apoptosis. While apoptosis is typically regarded as a controlled, non-inflammatory process that facilitates cellular clearance via phagocytosis [90]. Failure to remove apoptotic cells can lead to secondary necrosis. Via caspase-3-mediated GSDME cleavage that forms a cytotoxic N-terminal p30 fragment [91]. This fragment disrupts the plasma membrane integrity, resulting in membrane permeabilization and a shift from apoptosis to a pro-inflammatory form of cell death [91].

Here, we showed that CP-induced apoptosis, caspase-3 activation, and GSDME cleavage were all inhibited by VBIT-4 and VBIT-12 (Fig. 5A–D). Similarly, VDAC1 oligomerization mediated mtDNA release [43, 44], which activates the NLRP3 inflammasome, caspase-1, and pyroptosis via GSDMD cleavage (Fig. 5). VBIT-4 effectively inhibited NLRP3 assembly, caspase-1/GSDMD activation in cultured cells (Fig. 5) as well as in AD-like and DSS-induced colitis models (Figs. 6F, G, 7C, E).

Thus, VDAC1 overexpression and oligomerization underlies apoptosis, secondary necrosis, and pyroptosis via caspase-3 and caspase-1 activation, respectively (Fig. 8A), with GSDME and GSDMD cleavage as key downstream events [45, 72, 92, 93]. VDAC1 interacting compounds VBIT-4 and VBIT-12 blocked these processes (Figs. 1, 3, 5). Furthermore, the chemical FOS promotes NLRP3 inflammasome activation through VDAC1 oligomerization [94] and HK dissociation from the mitochondria, hereby triggering inflammasome assembly and activation [45] (discussed below).

Ferroptosis, an iron-dependent form of PCD, is characterized by an accumulation of lipid peroxides. The mitochondria play a crucial role in this process as ROS generators, and this PCD, which is associated with morphological mitochondrial changes [27, 28, 30, 95, 96], is also regulated by VDAC1 (Fig. 8B). The role of VDAC1 in regulating ferroptosis has been demonstrated in several studies, including reports showing that inhibition of VDAC1 oligomerization blocks cysteine deprivation–induced ferroptosis [97, 98].

Erastin and APAP induce VDAC1 expression/oligomerization, Cyto *c* release, and ferroptosis (Fig. 8B). VBIT-4/12 prevents these by reducing ROS and lipid peroxidation, as shown via calcein (Fe^2^⁺), C11-BODIPY (lipid ROS), and GPX4 monitoring (Figs. 4, 6K, L, 7E, G).

As depicted in Fig. 4B, erastin inhibits the system Xc- that exchanges intracellular glutamate for extracellular cystine. The cystine is then converted to cysteine in the cytosol, which is used to produce GSH, a cofactor for GPX4. GSH depletion increases ROS, leading to lipid peroxidation. Fe^3+^ enters through the transferrin receptor and is converted to Fe^2+^ in the endosome. Once released into the cytosol, it undergoes Fenton chemistry, generating ROS that further drive lipid peroxidation.

Both erastin and APAP [49] (Figs. 3, 4) induce VDAC1 overexpression and oligomerization, releasing Cyto *c*, impairing electron transport and enhancing ROS production (Fig. 8B) [63]. ROS exit the mitochondria through VDAC1 oligomers, elevating cellular ROS and promoting ferroptosis. Using calcein (to monitor iron) and C11-BODIPY (for lipid peroxidation), and GPX4 expression as a ferroptosis marker, we showed that both compounds suppress erastin- and APAP-induced ferroptosis that is inhibited by VBIT-4 and VBIT-12 (Figs. 4, 6K, L, 7E, G).

Together, these findings demonstrate a clear connection between VDAC1 overexpression, its oligomerization, and the activation of apoptosis, secondary necrosis, pyroptosis, and ferroptosis—all of which are effectively inhibited by VBIT-4 and VBIT-12 (Fig. 8).

### VBIT-4 and VBIT-12 prevent HK-I and HK-II detachment from mitochondrial VDAC1

HKs are multifunctional proteins that regulate glucose metabolism, antioxidant function, and cell death [99]. The binding of HK I and HK-II to VDAC1 [45, 53, 62, 100–104] is a well-studied mechanism involved in metabolic regulation, cell survival, and apoptosis inhibition, especially in tissues with high energy demand like brain and muscle.

Using the PLA assay, we demonstrated that HK-II interacts with VDAC1 under physiological conditions, and that this interaction is disrupted upon CP-induced apoptosis. This dissociation is prevented by VBIT-4 and VBIT-12 (Fig. 2B), consistent with previous findings [63]. Structural and biochemical evidence supports a 1:1 binding ratio between HK and VDAC1. Furthermore, the observation that HK dissociation promotes VDAC1 oligomerization [45], suggests that inhibition of VDAC1 oligomerization enhances HK binding to VDAC1, as shown in our study.

Their function depends on their subcellular localization: When bound to mitochondria via VDAC1, HKs promote glycolysis and cell survival with their detached, contribute to apoptosis [53, 100, 102–104]. The subcellular localization of HKs varies in different disease states [105–107]. HK–VDAC1 also interactions vary across disease states and have been linked to cancer, neurodegeneration, and psychiatric disorders. Thus, these findings led to the search for small molecules and peptides that can disrupt or stabilize this complex [53, 100, 101]. In cancer, elevated mitochondrially-bound HK-I and HK-II result in a high rate of glycolysis and lactate production, a metabolic support for the Warburg effect [15, 108], promoting tumor growth while inhibiting an apoptosis. Therefore, disrupting the HK–VDAC1 complex, with their detachment enabling the activation of apoptosis [53, 100, 102–104], has emerged as a potential anti-cancer strategy.

In neurons, HK and glycolysis are involved in synapse formation, and neurite growth, as well as in astrocyte, oligodendrocyte, and microglia activation. These processes are also implicated in brain aging and neurodegenerative diseases [109].

In mood and psychiatric disorders, reduced mitochondrial HK-I was observed in the brains of patients with depression, bipolar disorder, and schizophrenia, possibly contributing to impaired energy metabolism and increased oxidative stress, and disrupting brain growth and development [105].

In amyotrophic lateral sclerosis (ALS), a reduction in HK-I concentration in the spinal cord promotes VDAC1–mutant SOD1 interactions, triggering mitochondrial dysfunction and neuronal death [106]. Similarly, Aβ causes HK-I detachment, reducing ATP, increasing ROS, and inducing neuronal death [40, 107].

VSTM2L has been shown to stabilize the VDAC1–HK-II interaction and prevent ferroptosis by inhibiting VDAC1 oligomerization [110]. Moreover, HK detachment from the mitochondria also promotes NLRP3 inflammasome assembly and activation [45], highlighting the tight link between HK release, cell death, and disease.

Taken together, the dissociation of HK from the mitochondria is closely linked to the activation of cell death and/or inflammatory pathways, contributing to the development of various diseases. Therefore, preventing HK detachment from VDAC1 could serve as a promising therapeutic strategy for treating diseases that manifest this event.

Although several inhibitors of HK activity have been developed to disrupt glycolysis [100, 111], they also affect tissues like the brain, retina, and testes which rely on glucose as their primary energy source. Despite these concerns, targeting the VDAC1–HK interaction has emerged as a promising target for anti-cancer therapy [111, 112]. Several agents such as HK-I–derived peptides [113], clotrimazole [114], methyl jasmonate [100], and VDAC1-based peptides [53] induce HK detachment and promote apoptosis.

In contrast, VBIT-4 and VBIT-12 prevent HKI/II detachment induced by agents like selenite [63], Aβ [40, 107], and, as demonstrated here for CP via PLA for VDAC1 and HK-II, and by monitoring HK-I-GFP localization (Fig. 2D). Because VBIT-4 and VBIT-12 are the only known compounds that prevent HK detachment, they are promising candidates for treating diseases that are linked to HK detachment.

### VBIT-4 and VBIT-12 protect against programmed cell death and associated diseases

VBIT-4 and VBIT-12 offer protection against apoptosis, secondary necrosis, pyroptosis, ferroptosis, and related diseases, targeting PCD forms implicated in cancer, hematologic and autoimmune disorders, neurodegeneration, and infections (Table 1) [4–11].

Mitochondrial dysfunction is a central feature of AD, as evidenced by altered mitochondrial morphology, biogenesis, enzyme activity, oxidative stress, and metabolism [115], with metabolic defects in AD patients developing many decades before dementia symptoms appear [116, 117].

Using the 5XFAD mouse model of AD mouse model, we we previously demonstrated that VDAC1 is overexpressed around Aβ plaques, contributing to neuronal loss, neuroinflammation, and metabolic impairment [46] VBIT-4 effectively protected against AD-associated pathophysiological changes and prevented cognitive decline [46, 73] (Fig. 6). Similar VDAC1 upregulation was seen in PC-12 cells treated with Aβ1-42 and in APP/PS1 mice [73].

Here, we showed that VBIT-4 not only prevented neuronal loss, reduced neuroinflammation, improved astroglial and microglial cell morphology, and increased microglial activation (Fig. 6, Table S3), but also inhibited pyroptosis and ferroptosis (Fig. 6F–L), both of which are associated with AD [6]. Elevated GSDMD levels were found in the cortex of the 5XFAD mice, but not in those treated with VBIT-4 (Fig. 6F,G). This suggests that VBIT-4 treatment may have mitigated the inflammation observed in these mice, as reflected by decreased levels of NF-κB, cytokines, IL-1β, TNF-α, and NLRP3—a key component of the inflammasome associated with neuroinflammation in AD [46, 118, 119]. Pyroptosis and its associated inflammation are linked to various diseases, including inflammatory conditions [7, 8], cancers, and central nervous system disorders [6].

Similarly, the elevation of activated caspase-3 and GSDME levels in the 5XFAD mice were not observed when the mice were treated with VBIT-4 (Fig. 6H–J), suggesting that VBIT-4 also protects against apoptosis-driven secondary necrosis or GSDME-mediated pyroptosis [91]. Rotenone and 6-hydroxydopamine induced GSDME-mediated pyroptosis in mice and human neurons [120].

Glycated Aβ (gAβ) was recently shown to trigger mtDNA release and to activate the cGAS-STING pathway in AD in a VDAC1-dependent manner [121], consistent with previous reports [43, 44]. Also, the pollutant perfluorooctane sulfonate was shown to elevate VDAC1 oligomerization and NLRP3 inflammasome activation—effects that were alleviated by VBIT-12 [94].

We further showed that ferroptosis, as reflected by increased lipid peroxidation (C11-BODIPY staining) and reduced GPX4 levels that occurred in the 5XFAD mice, was prevented by VBIT-4 (Fig. 6K, L). Similarly, decreased GPX4 expression was observed in PC-12 cells treated with Aβ1-42, but not in those with silenced VDAC1 expression, and in an APP/PS1 AD-mouse model that was treated with VBIT-4 [73].

In a previous study of DSS-induced colitis [47], which is associated with mitochondrial dysfunction [122], VDAC1 was overexpressed and VBIT-12 treatment reduced inflammatory cell infiltration, apoptosis, mtDNA release, caspase-1 activation, and NLRP3 signaling. Here, we demonstrated that apoptosis, pyroptosis, secondary necrosis, and ferroptosis were also activated in this colitis mouse model (Fig. 6), but were prevented in the VBIT-12-treated mice [47].

Ferroptosis has been implicated in the pathogenesis of many diseases such as mitochondrial disorders, liver and renal diseases, cardiomyopathy [73, 96], and neurodegenerative disorders [123].

A hallmark of many neurodegenerative diseases is iron accumulation in the brain [124], and ferroptosis-related genes are notably dysregulated in astrocytes of the AD entorhinal cortex [125].

Various drugs and compounds are being explored or used to treat ferroptosis-related conditions, either by directly targeting the mechanisms underlying ferroptosis or by modulating related pathways. These include ferroptosis inhibitors (ferrostatin-1, liproxstatin-1), antioxidants, and redox modulators (N-acetylcysteine, vitamins E and C), and iron chelators (desferoxamine, deferiprone). While these inhibitors do not directly inhibit ferroptosis, they act indirectly by scavenging lipid peroxyl radicals, reducing lipid peroxide accumulation, or modulating oxidative stress. In contrast, VBIT-4 and VBIT-12 prevent ferroptosis by targeting the mitochondria/VDAC1, as shown here (Figs. 3, 4). This is further supported by the findings that VBIT-12 or VBIT-4, by inhibiting VDAC1 oligomerization, protected against ferroptosis in APAP-induced liver injury in mice [49] and in an APP/PS1 mouse AD model [73].

## Conclusions

VDAC1, a multi-functional mitochondrial protein, when overexpressed and oligomerized, plays a central and critical role in the processes of apoptosis, secondary necrosis, ferroptosis, and pyroptosis, contributing to a wide range of diseases (Table 1, Fig. 8). By preventing VDAC1 oligomerization, VBIT-4 and VBIT-12 mitigate mitochondrial dysfunction, PCD, mtDNA release, HK detachment, inflammation, and associated diseases [43–45].

The various PCD forms are interconnected, and blocking one pathway often activates others [1–3]. Here, we showed that VBIT-4 or VBIT-12 are the first compounds to effectively block the known forms of PCD in cultured cells and disease models by targeting VDAC1 oligomerization, and they can be viewed as a single molecule, with a single target. Unlike drugs, such as metformin, which act via multiple pathways to target cancer, obesity, and liver, cardiovascular, and renal diseases [126], VBIT4 or VBIT-12 offer a single-target strategy with broad therapeutic potential.

Our findings outline key events linking VDAC1 to disease: (a) its overexpression and oligomerization are common in many pathologies, (b) these diseases are associated with mitochondria-driven PCD and inflammation, (c) targeting VDAC1 oligomerization represents a novel therapeutic approach, and (d) VBIT-4 and VBIT-12, by inhibiting VDAC1 oligomerization, inhibit mitochondria-associated PCD and inflammation, and improved disease-related pathologies in AD and colitis animal models. These findings suggest that VDAC1 could bridge mitochondrial dysfunction, PCD, and inflammation across a range of diseases (Table 1).

Finally, VBIT-4 and VBIT-12 are the first compounds shown to inhibit multiple forms of PCD by preventing VDAC1 oligomerization, underscoring their potential for treating diseases associated with HK detachment, cell death, and inflammation.

## Supplementary Information

Below is the link to the electronic supplementary material.

Supplementary Material 1

## Data Availability

No datasets were generated or analysed during the current study.

## References

[1] Bertheloot D, Latz E, Franklin BS (2021) Necroptosis, pyroptosis and apoptosis: an intricate game of cell death. Cell Mol Immunol 18:1106–1121 33785842 10.1038/s41423-020-00630-3 PMC8008022

[2] Snyder AG, Oberst A (2021) The antisocial network: cross talk between cell death programs in host defense. Annu Rev Immunol 39:77–101 33441019 10.1146/annurev-immunol-112019-072301 PMC8594462

[3] Tang D, Kang R, Berghe TV, Vandenabeele P, Kroemer G (2019) The molecular machinery of regulated cell death. Cell Res 29:347–364 30948788 10.1038/s41422-019-0164-5 PMC6796845

[4] Wang Y, Li H, He Q, Zou R, Cai J, Zhang L (2024) Ferroptosis: underlying mechanisms and involvement in neurodegenerative diseases. Apoptosis 29:3–21 37848673 10.1007/s10495-023-01902-9

[5] Stockwell BR, Friedmann Angeli JP, Bayir H et al (2017) Ferroptosis: a regulated cell death nexus linking metabolism, redox biology, and disease. Cell 171:273–285 28985560 10.1016/j.cell.2017.09.021 PMC5685180

[6] Hu Y, Wang B, Li S, Yang S (2022) Pyroptosis, and its role in central nervous system disease. J Mol Biol 434:167379 34838808 10.1016/j.jmb.2021.167379

[7] Xu B, Jiang MZ, Chu Y et al (2018) Gasdermin D plays a key role as a pyroptosis executor of non-alcoholic steatohepatitis in humans and mice. J Hepatol 68:773–782 29273476 10.1016/j.jhep.2017.11.040

[8] Ma C, Yang D, Wang B et al (2020) Gasdermin D in macrophages restrains colitis by controlling cGAS-mediated inflammation. Sci Adv 6:eaaz6717 32671214 10.1126/sciadv.aaz6717 PMC7314554

[9] Han C, Liu Y, Dai R, Ismail N, Su W, Li B (2020) Ferroptosis and its potential role in human diseases. Front Pharmacol 11:239 32256352 10.3389/fphar.2020.00239 PMC7090218

[10] Shoshan-Barmatz V, Shteinfer-Kuzmine A, Verma A (2020) VDAC1 at the intersection of cell metabolism, apoptosis, and diseases. Biomolecules 10:1485 33114780 10.3390/biom10111485 PMC7693975

[11] Shen SK, Shao YA, Li CH (2023) Different types of cell death and their shift in shaping disease. Cell Death Discov 9:284 37542066 10.1038/s41420-023-01581-0 PMC10403589

[12] Li J, Cao F, Yin HL et al (2020) Ferroptosis: past, present and future. Cell Death Dis 11:88 32015325 10.1038/s41419-020-2298-2 PMC6997353

[13] Ashkenazi A (2015) Targeting the extrinsic apoptotic pathway in cancer: lessons learned and future directions. J Clin Invest 125:487–489 25642709 10.1172/JCI80420 PMC4319431

[14] Carneiro BA, El-Deiry WS (2020) Targeting apoptosis in cancer therapy. Nat Rev Clin Oncol 17:395–417 32203277 10.1038/s41571-020-0341-y PMC8211386

[15] Shoshan-Barmatz V, Ben-Hail D, Admoni L, Krelin Y, Tripathi SS (2015) The mitochondrial voltage-dependent anion channel 1 in tumor cells. Biochim Biophys Acta 1848:2547–2575 25448878 10.1016/j.bbamem.2014.10.040

[16] Biasutto L, Azzolini M, Szabò I, Zoratti M (2016) The mitochondrial permeability transition pore in AD 2016: an update. Biochimica et Biophysica Acta (BBA) - Mol Cell Res 1863:2515–2530 10.1016/j.bbamcr.2016.02.012 26902508

[17] Antignani A, Youle RJ (2006) How do Bax and Bak lead to permeabilization of the outer mitochondrial membrane? Curr Opin Cell Biol 18:685–689 17046225 10.1016/j.ceb.2006.10.004

[18] Keinan N, Tyomkin D, Shoshan-Barmatz V (2010) Oligomerization of the mitochondrial protein voltage-dependent anion channel is coupled to the induction of apoptosis. Mol Cell Biol 30:5698–5709 20937774 10.1128/MCB.00165-10 PMC3004265

[19] Weisthal S, Keinan N, Ben-Hail D, Arif T, Shoshan-Barmatz V (2014) Ca(2+)-mediated regulation of VDAC1 expression levels is associated with cell death induction. Biochim Biophys Acta 1843:2270–2281 24704533 10.1016/j.bbamcr.2014.03.021

[20] Yu P, Zhang X, Liu N, Tang L, Peng C, Chen X (2021) Pyroptosis: mechanisms and diseases. Signal Transduct Target Ther 6:128 33776057 10.1038/s41392-021-00507-5 PMC8005494

[21] Shi J, Zhao Y, Wang K et al (2015) Cleavage of GSDMD by inflammatory caspases determines pyroptotic cell death. Nature 526:660–665 26375003 10.1038/nature15514

[22] Rogers C, Fernandes-Alnemri T, Mayes L, Alnemri D, Cingolani G, Alnemri ES (2017) Cleavage of DFNA5 by caspase-3 during apoptosis mediates progression to secondary necrotic/pyroptotic cell death. Nat Commun 8:14128 28045099 10.1038/ncomms14128 PMC5216131

[23] Yu J, Li S, Qi J et al (2019) Cleavage of GSDME by caspase-3 determines lobaplatin-induced pyroptosis in colon cancer cells. Cell Death Dis 10:193 30804337 10.1038/s41419-019-1441-4 PMC6389936

[24] De Schutter E, Ramon J, Pfeuty B et al (2021) Plasma membrane perforation by GSDME during apoptosis-driven secondary necrosis. Cell Mol Life Sci 79:19 34971436 10.1007/s00018-021-04078-0 PMC8720079

[25] Bergsbaken T, Fink SL, Cookson BT (2009) Pyroptosis: host cell death and inflammation. Nat Rev Microbiol 7:99–109 19148178 10.1038/nrmicro2070 PMC2910423

[26] Hou J, Zhao R, Xia W et al (2020) PD-L1-mediated gasdermin C expression switches apoptosis to pyroptosis in cancer cells and facilitates tumour necrosis. Nat Cell Biol 22:1264–1275 32929201 10.1038/s41556-020-0575-z PMC7653546

[27] Oh SJ, Ikeda M, Ide T, Hur KY, Lee MS (2022) Mitochondrial event as an ultimate step in ferroptosis. Cell Death Discov 8:422 36209144 10.1038/s41420-022-01199-8 PMC9547870

[28] Liu Y, Lu S, Wu LL, Yang L, Yang L, Wang J (2023) The diversified role of mitochondria in ferroptosis in cancer. Cell Death Dis 14:519 37580393 10.1038/s41419-023-06045-y PMC10425449

[29] Xie Y, Hou W, Song X et al (2016) Ferroptosis: process and function. Cell Death Differ 23:369–379 26794443 10.1038/cdd.2015.158 PMC5072448

[30] Gao M, Yi J, Zhu J et al (2019) Role of mitochondria in ferroptosis. Mol Cell 73(354–363):e353 10.1016/j.molcel.2018.10.042 PMC6338496 30581146

[31] Han D, Antunes F, Canali R, Rettori D, Cadenas E (2003) Voltage-dependent anion channels control the release of the superoxide anion from mitochondria to cytosol. J Biol Chem 278:5557–5563 12482755 10.1074/jbc.M210269200

[32] Tang D, Chen X, Kang R, Kroemer G (2021) Ferroptosis: molecular mechanisms and health implications. Cell Res 31:107–125 33268902 10.1038/s41422-020-00441-1 PMC8026611

[33] Patane GT, Putaggio S, Tellone E et al (2023) Ferroptosis: emerging role in diseases and potential implication of bioactive compounds. Int J Mol Sci. 10.3390/ijms242417279 38139106 10.3390/ijms242417279 PMC10744228

[34] Malireddi RKS, Bynigeri RR, Mall R, Connelly JP, Pruett-Miller SM, Kanneganti TD (2023) Inflammatory cell death, PANoptosis, screen identifies host factors in coronavirus innate immune response as therapeutic targets. Commun Biol 6:1071 37864059 10.1038/s42003-023-05414-9 PMC10589293

[35] Malireddi RKS, Kesavardhana S, Kanneganti TD (2019) ZBP1 and TAK1: master regulators of NLRP3 inflammasome/pyroptosis, apoptosis, and necroptosis (PAN-optosis). Front Cell Infect Microbiol 9:406 31850239 10.3389/fcimb.2019.00406 PMC6902032

[36] Sun X, Yang Y, Meng X, Li J, Liu X, Liu H (2024) PANoptosis: mechanisms, biology, and role in disease. Immunol Rev 321:246–262 37823450 10.1111/imr.13279

[37] Shi D, Bai Y, Long R et al (2025) Neuronal LAMP2A-mediated reduction of adenylyl cyclases induces acute neurodegenerative responses and neuroinflammation after ischemic stroke. Cell Death Differ 32:337–352 39341961 10.1038/s41418-024-01389-0 PMC11802923

[38] Monzel AS, Enríquez JA, Picard M (2023) Multifaceted mitochondria: moving mitochondrial science beyond function and dysfunction. Nat Metab 5:546–562 37100996 10.1038/s42255-023-00783-1 PMC10427836

[39] Geula S, Naveed H, Liang J, Shoshan-Barmatz V (2012) Structure-based analysis of VDAC1 protein: defining oligomer contact sites. J Biol Chem 287:2179–2190 22117062 10.1074/jbc.M111.268920 PMC3265896

[40] Smilansky A, Dangoor L, Nakdimon I, Ben-Hail D, Mizrachi D, Shoshan-Barmatz V (2015) The voltage-dependent anion channel 1 mediates amyloid beta toxicity and represents a potential target for Alzheimer disease therapy. J Biol Chem 290:30670–30683 26542804 10.1074/jbc.M115.691493 PMC4692199

[41] Shoshan-Barmatz V, Maldonado EN, Krelin Y (2017) VDAC1 at the crossroads of cell metabolism, apoptosis and cell stress. Cell Stress 1:11–36 30542671 10.15698/cst2017.10.104 PMC6287957

[42] Shoshan-Barmatz V, Krelin Y, Shteinfer-Kuzmine A, Arif T (2017) Voltage-dependent anion channel 1 as an emerging drug target for novel anti-cancer therapeutics. Front Oncol 7:154 28824871 10.3389/fonc.2017.00154 PMC5534932

[43] Kim J, Gupta R, Blanco LP et al (2019) VDAC oligomers form mitochondrial pores to release mtDNA fragments and promote lupus-like disease. Science 366:1531–1536 31857488 10.1126/science.aav4011 PMC8325171

[44] Riley JS, Tait SW (2020) Mitochondrial DNA in inflammation and immunity. EMBO Rep 21:e49799 32202065 10.15252/embr.201949799 PMC7132203

[45] Baik SH, Ramanujan VK, Becker C, Fett S, Underhill DM, Wolf AJ (2023) Hexokinase dissociation from mitochondria promotes oligomerization of VDAC that facilitates NLRP3 inflammasome assembly and activation. Sci Immunol 8:eade7652 37327321 10.1126/sciimmunol.ade7652 PMC10360408

[46] Verma A, Shteinfer-Kuzmine A, Kamenetsky N et al (2022) Targeting the overexpressed mitochondrial protein VDAC1 in a mouse model of Alzheimer’s disease protects against mitochondrial dysfunction and mitigates brain pathology. Transl Neurodegener 11:58 36578022 10.1186/s40035-022-00329-7 PMC9795455

[47] Verma A, Pittala S, Alhozeel B et al (2022) The role of the mitochondrial protein VDAC1 in inflammatory bowel disease: a potential therapeutic target. Mol Ther 30(2):726–744 34217890 10.1016/j.ymthe.2021.06.024 PMC8821898

[48] Zhang E, Al-Amily I, Mohammed S et al (2019) Preserving insulin secretion in diabetes by inhibiting VDAC1 overexpression and surface translocation in β cells. Cell Metabol 29(1):64–77 10.1016/j.cmet.2018.09.008 PMC6331340 30293774

[49] Niu B, Lei X, Xu Q et al (2022) Protecting mitochondria via inhibiting VDAC1 oligomerization alleviates ferroptosis in acetaminophen-induced acute liver injury. Cell Biol Toxicol 38(3):505–530 34401974 10.1007/s10565-021-09624-x

[50] Paschon V, Morena BC, Correia FF et al (2019) VDAC1 is essential for neurite maintenance and the inhibition of its oligomerization protects spinal cord from demyelination and facilitates locomotor function recovery after spinal cord injury. Sci Rep 9:14063 31575916 10.1038/s41598-019-50506-4 PMC6773716

[51] Thompson EA, Cascino K, Ordonez AA et al (2021) Metabolic programs define dysfunctional immune responses in severe COVID-19 patients. Cell Rep 34:108863 33691089 10.1016/j.celrep.2021.108863 PMC7908880

[52] Di Domizio J, Gulen MF, Saidoune F et al (2022) The cGAS-STING pathway drives type I IFN immunopathology in COVID-19. Nature 603:145–151 35045565 10.1038/s41586-022-04421-w PMC8891013

[53] Arzoine L, Zilberberg N, Ben-Romano R, Shoshan-Barmatz V (2009) Voltage-dependent anion channel 1-based peptides interact with hexokinase to prevent its anti-apoptotic activity. J Biol Chem 284:3946–3955 19049977 10.1074/jbc.M803614200

[54] Dai Z, Zhang W, Zhou L, Huang J (2023) Probing lipid peroxidation in ferroptosis: emphasizing the utilization of C11-BODIPY-based protocols. Methods Mol Biol 2712:61–72 37578696 10.1007/978-1-0716-3433-2_6

[55] Gustafsdottir SM, Schallmeiner E, Fredriksson S et al (2005) Proximity ligation assays for sensitive and specific protein analyses. Anal Biochem 345:2–9 15950911 10.1016/j.ab.2005.01.018

[56] Tajeddine N, Galluzzi L, Kepp O et al (2008) Hierarchical involvement of Bak, VDAC1 and Bax in cisplatin-induced cell death. Oncogene 27:4221–4232 18362892 10.1038/onc.2008.63

[57] Yang Z, Schumaker LM, Egorin MJ, Zuhowski EG, Guo Z, Cullen KJ (2006) Cisplatin preferentially binds mitochondrial DNA and voltage-dependent anion channel protein in the mitochondrial membrane of head and neck squamous cell carcinoma: possible role in apoptosis. Clin Cancer Res 12:5817–5825 17020989 10.1158/1078-0432.CCR-06-1037

[58] Castagna A, Antonioli P, Astner H et al (2004) A proteomic approach to cisplatin resistance in the cervix squamous cell carcinoma cell line A431. Proteomics 4:3246–3267 15378690 10.1002/pmic.200400835

[59] Karunanithi Nivedita A, Santhanam M, Babu V, Shteinfer-Kuzmine, A.,, Garcia Venzor A, Toiber D, Shoshan-Barmatz V. (2025) Signaling Pathways Regulating VDAC1 Overexpression Associated with Apoptosis, Pyroptosis, and Ferroptosis Cell communication revised version submitted. 10.1186/s12964-025-02647-5 PMC12910870 41572303

[60] Zhou L, Zhang YF, Yang FH, Mao HQ, Chen Z, Zhang L (2021) Mitochondrial DNA leakage induces odontoblast inflammation via the cGAS-STING pathway. Cell Commun Signal 19:58 34016129 10.1186/s12964-021-00738-7 PMC8136190

[61] Wang J-N, Wei T-T, Qiu Z-Y et al (2025) VDAC1 oligomerization activates PANoptosis via mtDNA-STING axis in age-related macular degeneration. Free Radical Biol Med 142:150–166

[62] Mathupala SP, Ko YH, Pedersen PL (2006) Hexokinase II: cancer’s double-edged sword acting as both facilitator and gatekeeper of malignancy when bound to mitochondria. Oncogene 25:4777–4786 16892090 10.1038/sj.onc.1209603 PMC3385868

[63] Ben-Hail D, Begas-Shvartz R, Shalev M et al (2016) Novel compounds targeting the mitochondrial protein VDAC1 inhibit apoptosis and protect against mitochondrial dysfunction. J Biol Chem 291:24986–25003 27738100 10.1074/jbc.M116.744284 PMC5122769

[64] Yagoda N, von Rechenberg M, Zaganjor E et al (2007) RAS-RAF-MEK-dependent oxidative cell death involving voltage-dependent anion channels. Nature 447:864–868 17568748 10.1038/nature05859 PMC3047570

[65] Hirayama T, Nagasawa H (2017) Chemical tools for detecting Fe ions. J Clin Biochem Nutr 60:39–48 28163381 10.3164/jcbn.16-70 PMC5281535

[66] Miotto G, Rossetto M, Di Paolo ML et al (2020) Insight into the mechanism of ferroptosis inhibition by ferrostatin-1. Redox Biol 28:101328 31574461 10.1016/j.redox.2019.101328 PMC6812032

[67] Parker JB, Griffin MF, Downer MA et al (2023) Chelating the valley of death: deferoxamine’s path from bench to wound clinic. Front Med (Lausanne). 10.3389/fmed.2023.1015711 36873870 10.3389/fmed.2023.1015711 PMC9975168

[68] Yamada N, Komada T, Ohno N, Takahashi M (2020) Acetaminophen-induced hepatotoxicity: different mechanisms of acetaminophen-induced ferroptosis and mitochondrial damage. Arch Toxicol 94:2255–2257 32236649 10.1007/s00204-020-02722-5

[69] Lamkanfi M, Kanneganti TD, Franchi L, Nunez G (2007) Caspase-1 inflammasomes in infection and inflammation. J Leukoc Biol 82:220–225 17442855 10.1189/jlb.1206756

[70] Lin Y, Li Z, Wang Y et al (2022) CCDC50 suppresses NLRP3 inflammasome activity by mediating autophagic degradation of NLRP3. EMBO Rep 23:e54453 35343634 10.15252/embr.202154453 PMC9066065

[71] Heneka MT, Golenbock DT, Latz E (2015) Innate immunity in Alzheimer’s disease. Nat Immunol 16:229–236 25689443 10.1038/ni.3102

[72] Liu H, Fan H, He P et al (2022) Prohibitin 1 regulates mtDNA release and downstream inflammatory responses. EMBO J 41:e111173 36245295 10.15252/embj.2022111173 PMC9753472

[73] Zhou XP, Tang XM, Li T et al (2023) IInhibition of VDAC1 rescues Aβ1-42-induced mitochondrial dysfunction and ferroptosis via activation of AMPK and Wnt/β-catenin pathways. Mediators Inflamm 2023:6739691 36816741 10.1155/2023/6739691 PMC9937775

[74] Wan H, Yan YD, Hu XM et al (2023) Inhibition of mitochondrial VDAC1 oligomerization alleviates apoptosis and necroptosis of retinal neurons following OGD/R injury. Ann Anat 247:152049 36690044 10.1016/j.aanat.2023.152049

[75] Wei SN, Zhang H, Lu Y et al (2023) Microglial voltage-dependent anion channel 1 signaling modulates sleep deprivation-induced transition to chronic postsurgical pain. Sleep 46:zsad039 36827092 10.1093/sleep/zsad039

[76] Shteinfer-Kuzmine A, Argueti-Ostrovsky S, Leyton-Jaimes MF et al (2022) Targeting the mitochondrial protein VDAC1 as a potential therapeutic strategy in ALS. Int J Mol Sci 23:9946 36077343 10.3390/ijms23179946 PMC9456491

[77] Luo XL, Yue J (2025) VDAC1 inhibition mitigates inflammatory status and oxidative stress in epileptic mice treated with the ketogenic diet. Neurochem Res. 10.1007/s11064-025-04366-2 40085179 10.1007/s11064-025-04366-2

[78] Jiang W, Du B, Chi Z et al (2007) Preliminary explorations of the role of mitochondrial proteins in refractory epilepsy: some findings from comparative proteomics. J Neurosci Res 85:3160–3170 17893921 10.1002/jnr.21384

[79] Belosludtsev KN, Serov DA, Ilzorkina AI et al (2023) Pharmacological and genetic suppression of VDAC1 alleviates the development of mitochondrial dysfunction in endothelial and fibroblast cell cultures upon hyperglycemic conditions. Antioxidants 12:1459 37507997 10.3390/antiox12071459 PMC10376467

[80] Tariq M, Sjogren M, Salehi A (2024) Sulindac prevents increased mitochondrial VDAC1 expression and cell surface mistargeting induced by pathological conditions in retinal cells. Biochem Biophys Res Commun 739:150558 39181068 10.1016/j.bbrc.2024.150558

[81] Mohammad Al-Amily I, Sjogren M, Duner P, Tariq M, Wollheim CB, Salehi A (2023) Ablation of GPR56 causes beta-cell dysfunction by ATP loss through mistargeting of mitochondrial VDAC1 to the plasma membrane. Biomolecules 13:557 36979492 10.3390/biom13030557 PMC10046417

[82] Torres-Odio S, Lei Y, Gispert S et al (2021) Loss of mitochondrial protease CLPP activates type I IFN responses through the mitochondrial DNA-cGAS-STING signaling axis. J Immunol 206:1890–1900 33731338 10.4049/jimmunol.2001016 PMC8026707

[83] Mingxing R, Ping H, Fengyi L et al (2024) Neutrophil airfreighter efficiently delivers siRNA-loaded nanocomplex to mononuclear phagocytes for inhibition of mtDNA-induced inflammation. Adv Funct Mater 35:2416336

[84] Wang DY, Li Y, Li GY et al (2024) Inhibition of PKC-δ retards kidney fibrosis via inhibiting cGAS-STING signaling pathway in mice. Cell Death Discov 10:314 38972937 10.1038/s41420-024-02087-z PMC11228024

[85] Klapper-Goldstein H, Verma A, Elyagon S et al (2020) VDAC1 in the diseased myocardium and the effect of VDAC1-interacting compound on atrial fibrosis induced by hyperaldosteronism. Sci Rep. 10.1038/s41598-020-79056-w 33328613 10.1038/s41598-020-79056-w PMC7744539

[86] Xia T, Yu JC, Chen Y, Chang X, Meng M (2024) Phosphoglycerate mutase 5 aggravates alcoholic liver disease through disrupting VDAC-1-dependent mitochondrial integrity. Int J Med Sci 21:755–764 38464835 10.7150/ijms.93171 PMC10920835

[87] Lv J, Zhang X, An X et al (2024) The inhibition of VDAC1 oligomerization promotes pigmentation through the CaMK-CRTCs/CREB-MITF pathway. Exp Cell Res 434:113874 38070860 10.1016/j.yexcr.2023.113874

[88] Dubinin MV, Stepanova AE, Mikheeva IB et al (2025) VBIT-4 rescues mitochondrial dysfunction and reduces skeletal muscle degeneration in a severe model of Duchenne muscular dystrophy. Int J Mol Sci. 10.3390/ijms26188845 41009414 10.3390/ijms26188845 PMC12469774

[89] Li Y, Cui J, Liu L et al (2024) mtDNA release promotes cGAS-STING activation and accelerated aging of postmitotic muscle cells. Cell Death Dis 15:523 39039044 10.1038/s41419-024-06863-8 PMC11263593

[90] Green DR, Oguin TH, Martinez J (2016) The clearance of dying cells: table for two. Cell Death Differ 23:915–926 26990661 10.1038/cdd.2015.172 PMC4987729

[91] De Schutter E, Roelandt R, Riquet FB, Van Camp G, Wullaert A, Vandenabeele P (2021) Punching holes in cellular membranes: biology and evolution of gasdermins. Trends Cell Biol 31:500–513 33771452 10.1016/j.tcb.2021.03.004

[92] Cullen SP, Kearney CJ, Clancy DM, Martin SJ (2015) Diverse activators of the NLRP3 inflammasome promote IL-1beta secretion by triggering necrosis. Cell Rep 11:1535–1548 26027935 10.1016/j.celrep.2015.05.003

[93] Wang YP, Gao WQ, Shi XY et al (2017) Chemotherapy drugs induce pyroptosis through caspase-3 cleavage of a gasdermin. Nature 547:99–103 28459430 10.1038/nature22393

[94] Ma Y, Yang W, Liang P et al (2024) The VDAC1 oligomerization regulated by ATP5B leads to the NLRP3 inflammasome activation in the liver cells under PFOS exposure. Ecotoxicol Environ Saf 281:116647 38944014 10.1016/j.ecoenv.2024.116647

[95] Mou Y, Wang J, Wu J et al (2019) Ferroptosis, a new form of cell death: opportunities and challenges in cancer. J Hematol Oncol 12:34 30925886 10.1186/s13045-019-0720-y PMC6441206

[96] Jiang X, Stockwell BR, Conrad M (2021) Ferroptosis: mechanisms, biology and role in disease. Nat Rev Mol Cell Biol 22:266–282 33495651 10.1038/s41580-020-00324-8 PMC8142022

[97] Zhou Q, Liu T, Qian W et al (2023) HNF4A-BAP31-VDAC1 axis synchronously regulates cell proliferation and ferroptosis in gastric cancer. Cell Death Dis 14:356 37296105 10.1038/s41419-023-05868-z PMC10256786

[98] Jang SK, Ahn SH, Kim G et al (2024) Inhibition of VDAC1 oligomerization blocks cysteine deprivation-induced ferroptosis via mitochondrial ROS suppression. Cell Death Dis 15:811 39521767 10.1038/s41419-024-07216-1 PMC11550314

[99] Rodriguez-Saavedra C, Morgado-Martinez LE, Burgos-Palacios A, King-Diaz B, Lopez-Coria M, Sanchez-Nieto S (2021) Moonlighting proteins: the case of the hexokinases. Front Mol Biosci 8:701975 34235183 10.3389/fmolb.2021.701975 PMC8256278

[100] Goldin N, Arzoine L, Heyfets A et al (2008) Methyl jasmonate binds to and detaches mitochondria-bound hexokinase. Oncogene 27:4636–4643 18408762 10.1038/onc.2008.108

[101] Gautier B, Foret Jacquard M, Guelfi S et al (2022) Mapping the N-terminal hexokinase-I binding site onto voltage-dependent anion channel-1 to block peripheral nerve demyelination. J Med Chem 65:11633–11647 35984330 10.1021/acs.jmedchem.2c00411

[102] Abu-Hamad S, Zaid H, Israelson A, Nahon E, Shoshan-Barmatz V (2008) Hexokinase-I protection against apoptotic cell death is mediated via interaction with the voltage-dependent anion channel-1: mapping the site of binding. J Biol Chem 283:13482–13490 18308720 10.1074/jbc.M708216200

[103] Azoulay-Zohar H, Israelson A, Abu-Hamad S, Shoshan-Barmatz V (2004) In self-defence: hexokinase promotes voltage-dependent anion channel closure and prevents mitochondria-mediated apoptotic cell death. Biochem J 377:347–355 14561215 10.1042/BJ20031465 PMC1223882

[104] Pastorino JG, Hoek JB (2008) Regulation of hexokinase binding to VDAC. J Bioenerg Biomembr 40:171–182 18683036 10.1007/s10863-008-9148-8 PMC2662512

[105] Regenold WT, Pratt M, Nekkalapu S, Shapiro PS, Kristian T, Fiskum G (2012) Mitochondrial detachment of hexokinase 1 in mood and psychotic disorders: implications for brain energy metabolism and neurotrophic signaling. J Psychiatr Res 46:95–104 22018957 10.1016/j.jpsychires.2011.09.018

[106] Magri A, Belfiore R, Reina S et al (2016) Hexokinase I N-terminal based peptide prevents the VDAC1-SOD1 G93A interaction and re-establishes ALS cell viability. Sci Rep 6:34802 27721436 10.1038/srep34802 PMC5056396

[107] Saraiva LM, Seixas da Silva GS, Galina A et al (2010) Amyloid-beta triggers the release of neuronal hexokinase 1 from mitochondria. PLoS ONE 5:e15230 21179577 10.1371/journal.pone.0015230 PMC3002973

[108] Hay N (2016) Reprogramming glucose metabolism in cancer: can it be exploited for cancer therapy? Nat Rev Cancer 16:635–649 27634447 10.1038/nrc.2016.77 PMC5516800

[109] da Mata LR, dos Santos LD, de Cerqueira CM (2023) Hexokinase and glycolysis: between brain cells life and death. Current Chem Biol 17:91–123

[110] Yang J, Lu X, Hao JL et al (2025) VSTM2L protects prostate cancer cells against ferroptosis via inhibiting VDAC1 oligomerization and maintaining mitochondria homeostasis. Nat Commun 16:1160 39880844 10.1038/s41467-025-56494-6 PMC11779845

[111] Krasnov GS, Dmitriev AA, Lakunina VA, Kirpiy AA, Kudryavtseva AV (2013) Targeting VDAC-bound hexokinase II: a promising approach for concomitant anti-cancer therapy. Expert Opin Ther Targets 17:1221–1233 23984984 10.1517/14728222.2013.833607

[112] S. G-K. (2015) Targeting Glycolytic Adaptations of Cancer Cells: From Molecular Mechanisms to Therapeutic Opportunities. In: Wondrak G (ed). Stress Response Pathways in Cancer, pp. 331–344.

[113] Gelb BD, Adams V, Jones SN, Griffin LD, MacGregor GR, McCabe ER (1992) Targeting of hexokinase 1 to liver and hepatoma mitochondria. Proc Natl Acad Sci U S A 89:202–206 1309605 10.1073/pnas.89.1.202 PMC48204

[114] Penso J, Beitner R (1998) Clotrimazole and bifonazole detach hexokinase from mitochondria of melanoma cells. Eur J Pharmacol 342:113–117 9544799 10.1016/s0014-2999(97)01507-0

[115] Onyango IG, Dennis J, Khan SM (2016) Mitochondrial dysfunction in Alzheimer’s disease and the rationale for bioenergetics based therapies. Aging Dis 7:201–214 27114851 10.14336/AD.2015.1007 PMC4809610

[116] Mosconi L (2005) Brain glucose metabolism in the early and specific diagnosis of Alzheimer’s disease. FDG-PET studies in MCI and AD. Eur J Nucl Med Mol Imaging 32:486–510 15747152 10.1007/s00259-005-1762-7

[117] Bhatia S, Rawal R, Sharma P, Singh T, Singh M, Singh V (2022) Mitochondrial dysfunction in Alzheimer’s disease: opportunities for drug development. Curr Neuropharmacol 20:675–692 33998995 10.2174/1570159X19666210517114016 PMC9878959

[118] Ising C, Venegas C, Zhang S et al (2019) NLRP3 inflammasome activation drives tau pathology. Nature 575:669–673 31748742 10.1038/s41586-019-1769-z PMC7324015

[119] Hanslik KL, Ulland TK (2020) The role of microglia and the Nlrp3 inflammasome in Alzheimer’s disease. Front Neurol 11:570711 33071950 10.3389/fneur.2020.570711 PMC7530640

[120] Neel DV, Basu H, Gunner G et al (2023) Gasdermin-E mediates mitochondrial damage in axons and neurodegeneration. Neuron 111:1222–1240 36917977 10.1016/j.neuron.2023.02.019 PMC10121894

[121] Akhter F, Akhter A, Schiff H, et al. (2024) Amyloid beta glycation leads to 1 neuronal mitochondrial dysfunction and Alzheimer’s pathogenesis through VDAC1-dependent mtDNA efflux. 10.1073/pnas.2505046122 PMC12664008 41248283

[122] Jackson DN, Theiss AL (2019) Gut bacteria signaling to mitochondria in intestinal inflammation and cancer. Gut Microbes 11:285–304 30913966 10.1080/19490976.2019.1592421 PMC7524274

[123] Yang Y, Jia X, Yang X et al (2024) Targeting VDAC: a potential therapeutic approach for mitochondrial dysfunction in Alzheimer’s disease. Brain Res 1835:148920 38599511 10.1016/j.brainres.2024.148920

[124] Lee S, Kovacs GG (2024) The irony of iron: the element with diverse influence on neurodegenerative diseases. Int J Mol Sci 25:4269 38673855 10.3390/ijms25084269 PMC11049980

[125] Dang Y, He Q, Yang S et al (2022) FTH1- and SAT1-induced astrocytic ferroptosis is involved in Alzheimer’s disease: evidence from single-cell transcriptomic analysis. Pharmaceuticals (Basel) 15:1177 36297287 10.3390/ph15101177 PMC9610574

[126] Lv ZQ, Guo YJ (2020) Metformin and its benefits for various diseases. Front Endocrinol 11:191 10.3389/fendo.2020.00191 PMC7212476 32425881

