# Supplementary material for: Targeting VDAC1 to protect against mitochondria-linked cell death pathways: apoptosis, pyroptosis, ferroptosis, and associated diseases
Source: Apoptosis. 2026 Apr 11;31(4):122. doi: 10.1007/s10495-025-02217-7 (PMC13070067; doi:10.1007/s10495-025-02217-7)
Supplement: Supplementary file 1 — Supplementary Material 1 [file 10495_2025_2217_MOESM1_ESM.pdf]

Supplementary Materials
Materials

Bovine serum albumin (BSA), dithiothreitol, leupeptin, cytochalasin B, sodium selenite, phenylmethylsulfonyl fluoride (PMSF), propidium iodide (PI), Triton X-100, Tween-20, hematoxylin, eosin, ferrostatin**,** acetaminophen (APAP), erastin, calcein-AM and ***cis*-**Diammineplatinum(II) dichloride (Cisplatin; CP) were obtained from Sigma (St. Louis, MO). Digitonin obtained from Calbiochem (Nottingham, UK). A TUNEL assay kit was obtained from Promega (Cat. No. G3250; Madison, WI), and paraformaldehyde was purchased from from Emsdiasum (Hatfield, PA). Dulbecco's Modified Eagle's Medium (DMEM), Roswell Park Memorial Institute (RPMI) 1640 medium, normal goat serum (NGS), fetal bovine serum (FBS), and the supplements L-glutamine and penicillin-streptomycin were from Gibco (Grand Island, NY). Ethylene glycol bis(succinimidyl succinate) (EGS) (Cat. No: TS-21565), MitoSOX red mitochondrial superoxide indicator (Cat. No.: M36008), Fluo-4, AM (Cat. No.: F14201), a GeneJET plasmid miniprep kit (Cat. No.: K0502) and C11-BODIPY™ 581/591 (Cat. No.: D3861) were obtained from Thermo Fisher Scientific (Waltham, MA). 3,3-diaminobenzidine (DAB) was obtained from ImmPact-DAB (Cat. No.: VE-SK-4105; Burlingame, CA). VBIT-4 and VBIT-12 were synthesized by ChemPartner Co. Ltd. Shanghai, China. Primary antibodies, their sources, and the dilutions used are detailed in Table S1. Horseradish peroxidase (HRP)-conjugated and fluorophore-conjugated secondary antibodies are also listed in Table S1.

dsDNA staining

Cells were permeabilized with 0.3% Triton X-100 in PBS and blocked for 2 h with blocking buffer containing 10% normal goat serum and 1% fatty acid-free BSA in PBS. Subsequently, cells were incubated for 3 h at room temperature with anti-dsDNA antibodies and anti-VDAC1 or anti-COX-IVc antibodies diluted in antibody solution (5% normal goat serum, 0.1% Triton X-100, and 1% fatty acid-free BSA in PBS). Following three washes with PBS, cells were incubated for 1 h at room temperature in the dark with fluorescent-conjugated secondary antibodies. After additional PBS washes, coverslips were mounted using Fluoroshield mounting medium (ImmunoBioScience, Mukilteo, WA). Slides were dried overnight at 4°C, and images were captured using a confocal microscope (Olympus IX81, Tokyo, Japan).

Cytochrome c release

HEK-293 cells treated with apoptosis inducer (selenite or cisplatin) in the absence or presence of VBIT-4 were harvested, washed twice with PBS, pH 7.4, gently resuspended at 6 mg/ml in ice cold buffer (100 mM KCl, 2.5 mM $MgCl_2$, 250 mM sucrose, 20 mM HEPES/KOH, pH 7.5, 0.2mM EDTA, 1mM dithiothreitol, 1μg/ml leupeptin, 5 mg/ml cytochalasin B, and 0.1 mM PMSF) containing 0.025% digitonin, and incubated for 10 min on ice. Samples were centrifuged at 10,000 g at 4 °C for 10 min to obtain supernatants (cytosolic extracts) and pellets (contains mitochondria). Cyto c released to the cytosol was analyzed by immunoblotting using Cyto c-specific antibodies. Anti-VDAC1 and anti-GAPDH antibodies were used to verify that the cytosolic extracts are free of mitochondria**.**

Protein extraction and quantification

Harvested cells were pelleted by centrifugation at 15,000 x g for 10 min at 4°C. Cell pellets were then re-suspended and incubated on ice for 30 min in lysis buffer (50 mM Tris-HCl, pH 7.5, 150 mM NaCl, 1mM EDTA, 1.5mM $MgCl_2$, 10% glycerol, 1% Triton-X100) supplemented with a protease inhibitor cocktail (Calbiochem, San Diego, CA). Cell lysates were centrifuged for 10 min at 15,000g at 4ºC, and the supernatant was analyzed for protein concentration using the Lowry method. The protein extracts were stored at -80ºC until further analyzed by SDS-gel electrophoresis and immunoblotting.

Gel electrophoresis and immunoblot analysis

Following SDS-PAGE, gels were either stained with Coomassie brilliant blue or used for immunoblotting with the selected antibodies. For immunostaining, membranes containing electro-transferred proteins were stained by Ponceau, washed with H_2O, and then incubated for 2h with tris buffered saline, pH 7.8 (TBST) containing 5% non-fat dry milk and 0.1% Tween-20. Subsequently, membranes were incubated with the desired primary antibodies, followed by intensive washing with TBST, and incubation with the appropriate secondary antibodies conjugated with HRP (Table S1). Enhanced chemiluminescent substrate (Advantsa, San Jose, CA) was used to detect HRP activity visualized by FUSION-FX (Vilber Lourmat) and bands intensities were quantified using Image J software (Sun Microsystems, CA).

Table S1. Antibodies used in the study

Antibodies against the specific protein, source, catalogue number, and the dilutions used in immunofluorescence/immunohistochemistry (IF/IHC) and immunoblot (WB) are presented.

Antibody	Source and Cat. No.	Dilution	
		IF/IHC	WB
Mouse monoclonal anti-TUBB3	Biolegend, San Diego, CA, 801202	-	1:500
Mouse monoclonal anti-IBA	Abcam, Cambridge, UK, ab283319	1:250	-
Mouse monoclonal anti-VDAC1	Abcam, Cambridge, UK, ab186321	1:750	-
Rabbit polyclonal anti-VDAC1	Abcam, Cambridge, UK, ab15895	1:500	1:5,000
Mouse monoclonal anti-GAPDH	Abcam, Cambridge, UK, ab9484	-	1:2,000
Mouse monoclonal anti-Cytochrome c	Abcam, Cambridge, UK, ab13575	-	1:2,000
Mouse monoclonal anti-actin	Millipore, Billerica, MA, MAB1501	-	1:40,000
Mouse monoclonal anti-TNFα	Abcam, Cambridge, UK, ab1793	1:300	1:3,000
Mouse monoclonal anti-GFAP	Santa Cruz Biotechnology, Texas, sc-33673	1:500	-
Mouse monoclonal anti-NLRP3/NALP3	Adipogen San Diego, CA, AG-20B-0014	1:500	-
Rabbit polyclonal anti-ASC	Proteintech, Chicago, IL, 10500-1-AP	1:500	-
Rabbit polyclonal anti-HK-II	Merck, New Jersey, AB3279	1:500	-
Rabbit monoclonal anti-cytochrome oxidase subunit IVc/COX6C	Abcam, Cambridge, UK, ab150422	1:400	-
Rabbit polyclonal Gasdermin E	Abcam, Cambridge, UK, ab215191	1:400	1:1,000
Rabbit monoclonal anti-cleaved Gasdermin D	Cell signaling technology, Danvers, Massachusetts, USA, 36425	1:400	-
Mouse monoclonal anti-caspase-1	Santa Cruz Biotechnology, Texas USA, sc392736	-	1:1,000
Rabbit polyclonal anti-IL1β	Abcam, Cambridge, UK, ab9722	1:200	
Rabbit monoclonal anti-Glutathione Peroxidase 4 (GPX4)	Abcam, Cambridge, UK, ab125066	1:500	1:5,000
Rabbit polyclonal anti-cleaved caspase 3	Cell Signaling, MA, 9661	1:400	-
Rabbit polyclonal anti-caspase 3	Invitrogen, CA, PA5 77887		1:2,000
Mouse monoclonal anti-dsDNA	Abcam, Cambridge, UK, ab27156	1:2,000	-
Anti-mouse IgG, Alexa Fluor 488	Abcam, Cambridge, UK, ab150113	1:1,000	-
Anti-mouse IgG, Alexa Fluor 555	Abcam, Cambridge, UK, ab150114	1:1,000	-
Anti-rabbit IgG, Alexa Fluor 555	Abcam, Cambridge, UK, ab150078	1:1,000	-
Anti-rabbit IgG, Alexa Fluor 488	Abcam, Cambridge, UK, ab150077	1:1,000	-
Goat anti-rabbit HRP	Promega, WI, W4018	-	1:15,000
Donkey anti-mouse HRP	Abcam, Cambridge, UK, ab98799	-	1:10,000

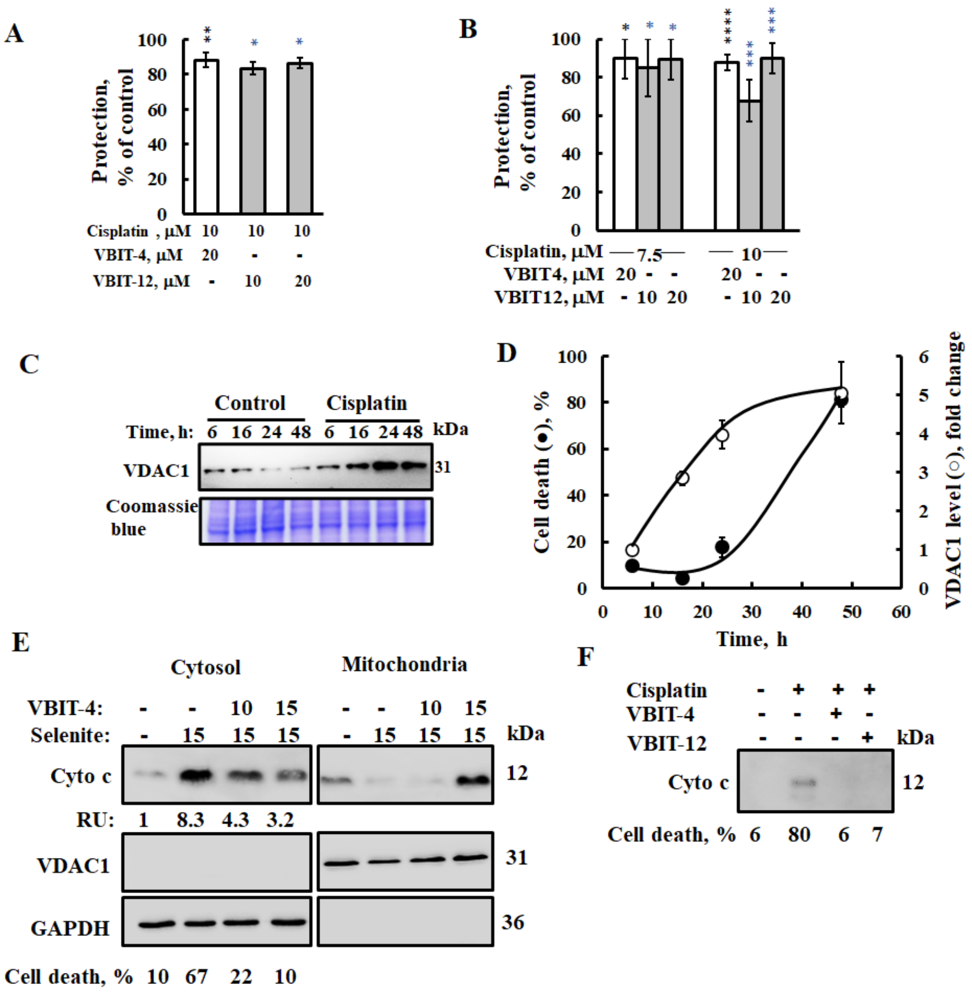

Fig. S1. VBIT-4 and VBIT-12 inhibit cisplatin-induced apoptosis

(A,B) HeLa cells were grown for 5 h in a serum-free medium, then pre-incubated (2 h) with the indicated concentration of VBIT-4 or VBIT-12, followed by incubation (48 h) with and without the indicated concentrations of CP. Cells were then subjected to apoptosis analysis using Annexin V-FITC/ PI staining (A) or cell death analysis using PI staining and a flow cytometry analysis (B). The percentages of cell death protection by VBIT-4 and VBIT 12 are shown. (C,D) HeLa cells were serum-starved for 5h and then incubated with or without 15µM of CP for 6, 12, 24 or 48h. Cells were harvested and subjected for VDAC1 expression by immunoblotting using specific antibodies (C). Coomassie blue is presented as a loading control. Cell death was assessed by PI staining followed by FACS analysis (D). For each time point, cisplatin-treated samples were compared with their respective time-matched controls.

(E) To assess Cyto c release, HEK-293 cells were pre-incubated with the indicated concentration of VBIT-4 for 2 h and then with or without selenite (15µM, 4 h). Then, cells were harvested and incubated on ice for 10 min with 0.025% digitonin, centrifuged (10,000g, 10 min), and the pellet (mitochondria) and supernatants (cytosol) were subjected to SDS-PAGE and immunoblotting, using anti-Cyto c, antibodies. Anti-VDAC1 and anti-GAPDH antibodies were used to verify that the cytosolic extracts are free of mitochondria. Quantitative analysis of selenite-induced Cyto c release to the cytosol done by ImageJ software and are presented as relative units (RU). (F) VBIT-4 and VBIT-12 protect against CP-induced cell death. HeLa cells were pre-incubated with 10 µM of VBIT-4 or VBIT-12, followed by incubation (48 h) with and without 15 µM of CP and cells were analyzed for Cyto c release as described in (E). Results represent the means ± SEM (n = 3), $^*p \leq 0.05$; $^{**}p \leq 0.01$; $^{***}p \leq 0.001$; $^{****}p \leq 0.0001$. Black and blue *p*-values indicate comparisons relative to the untreated control and CP-treated groups, respectively.

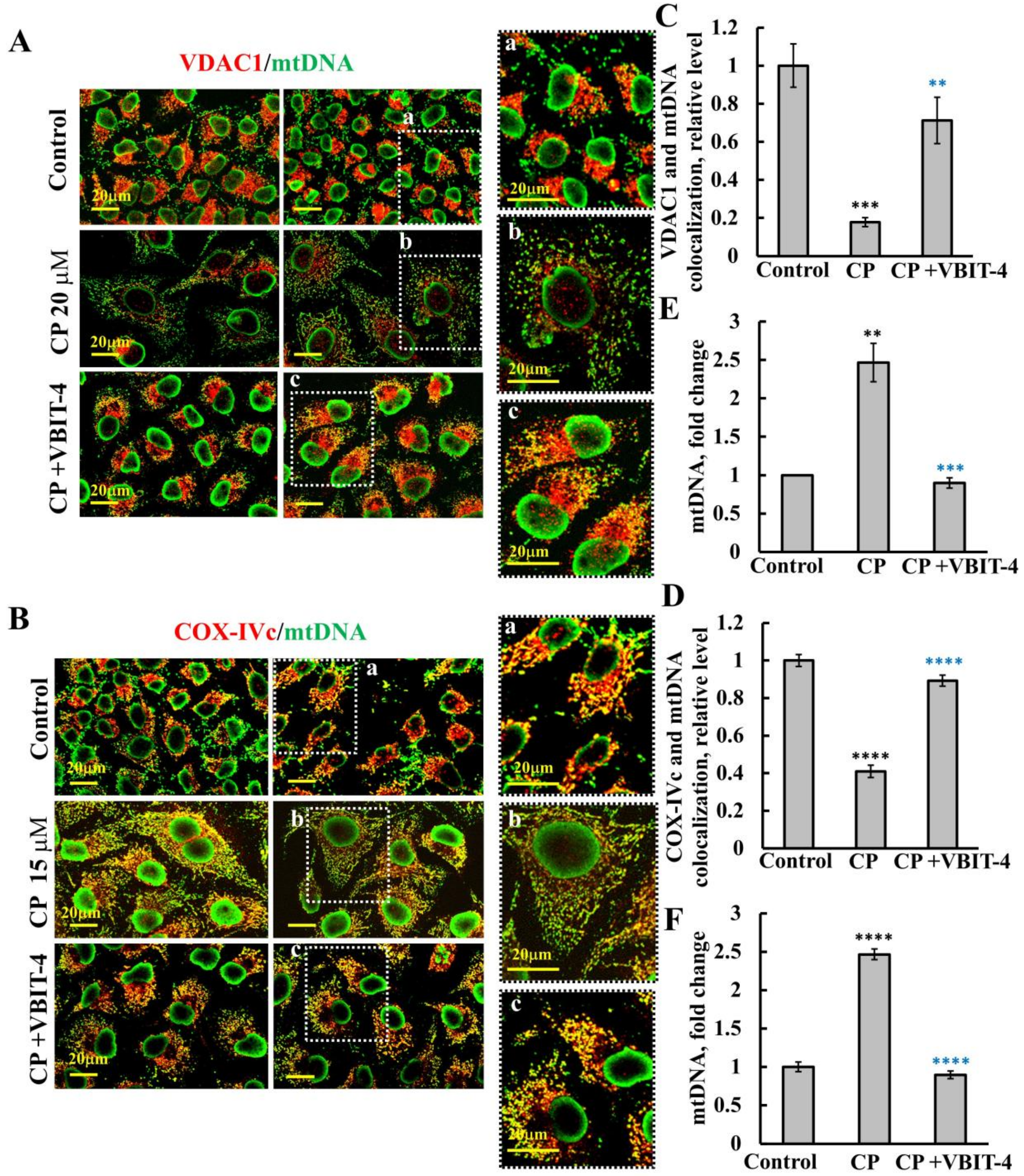

Fig. S2. VBIT-4 inhibits mtDNA release induced by cisplatin

HeLa cells were cultured on 13 mm glass coverslips and then pre-incubated with or without VBIT-4 (20 μM; 2 h), followed by cisplatin (CP, 15 or 20 μM; 48 h). Cells were then fixed with 4% paraformaldehyde and co-immunostained with anti–VDAC1 and anti-dsDNA antibodies (A) or anti-COX-VIc and anti-dsDNA antibodies (B) to label mitochondria and mtDNA, respectively and subsequently with the secondary antibodies. Cells were next washed with PBS containing 0.1% Triton and mounted on slides using Fluoroshield mounting medium. Cells were visualized using confocal microscopy. Representative images are shown (A,B) with enlargements (a,b,c). Red represents VDAC1 or COX-VIc and green dsDNA and yellow represents dsDNA co-localized with the mitochondria protein. (C,D) Quantification of dsDNA co-localization with VDAC1 (C) or with COX-IVc (D). (E,F) Quantification of dsDNA after subtraction the presence of dsDNA in the nucleus. Results represent the means ± SEM (n = 3), $^{**}p \leq 0.01$; $^{***}p \leq 0.001$; $^{****}p \leq 0.0001$. Black and blue *p*-values indicate comparisons relative to the untreated control and CP-treated groups, respectively.

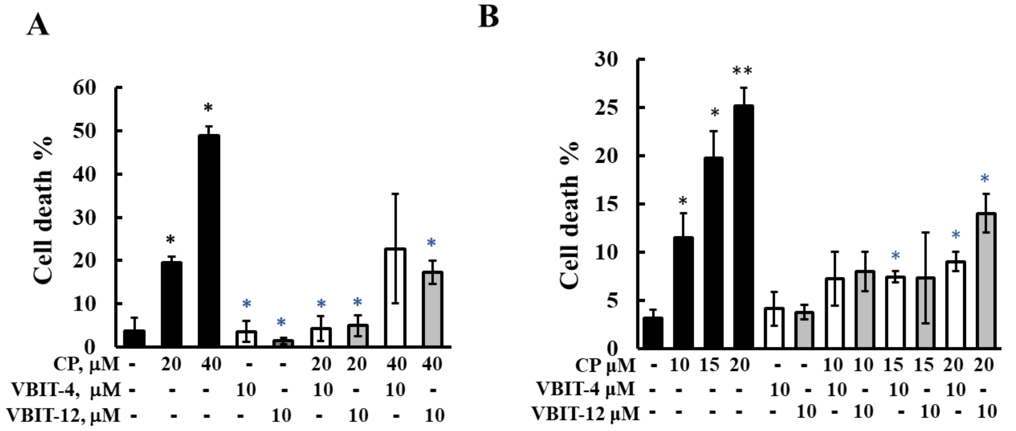

Fig. S3. VBIT-4 and VBIT-12 inhibit cisplatin-induced cell death
(A,B) These experiments were carried out parallel to the PLA assay in Figures 2B (A) and 5F (B), respectively. HeLa or PC-3 cells, respectively were pre-incubated with VBIT-4 or VBIT-12 (10 μM, 2 h), then incubated (48 h) with or without the indicated concentration of CP, followed by a cell death analysis using PI staining and flow cytometry. Results represent the means ± SEM (n = 3), *p ≤ 0.05; **p ≤ 0.01; ***p ≤ 0.001; ****p ≤ 0.0001. Black and blue *p*-values indicate comparisons relative to the untreated control and CP-treated groups, respectively

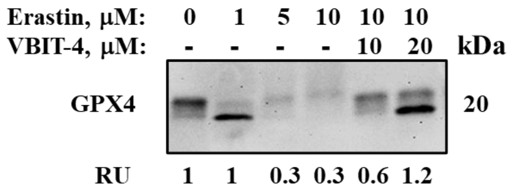

Fig. S4. VBIT-4 prevented erastin-induced reduction of GPX4 levels
C6 cells were preincubated with VBIT-4 (10 or 20 μM) for 2 hours, followed by incubation for 24 hours with or without the indicated concentrations of erastin. Cells were then harvested, lysed, and analyzed by SDS-PAGE and immunoblotting using an anti-GPX4 antibody. GPX4 expression levels were quantified using ImageJ software and are presented as relative units (RU).

Table S2. VDAC1 silencing reduced cisplatin-induced cell death

HeLa, A549, HepG2 or PC-3 cell lines were transfected with si-NT or with si-m/h-VDAC1-B (75 nM), using JetPrime transfection reagent, as described in the Methods section. At 24 h post transfection, cells were treated with different concentrations of cisplatin (5, 10, 15 and 20 μM) for 48 h and subjected to immunoblotting for VDAC1 expression levels using anti-VDAC1 specific antibodies and VDAC1 expression levels were quantified using ImageJ software. and are presented as % of the VDAC1 levels in the si-NT-transfected cells (control). Cell death induced by cisplatin in these was, determined using PI staining and a flow cytometer analysis. The concentrations of cisplatin required for induction of 50% cell death (IC_{50}) was determined for cells treated with si-NT or with si-m/h-VDAC1-B. The ratio between these IC_{50} is also presented.

**Cell line**	**Si-NT IC_{50}, μM**	**si-VDAC1- IC_{50}, μM**	**Fold increase in IC_{50}**	**VDAC1 level, % of control**
**HeLa**	8.1± 0.1	21.9 ± 0.2	2.7	10.2
**A549**	9.8 ± 0.2	23 ± 0.2	2.4	19.8
**HepG2**	7.1 ± 0.1	19.9 ± 0.1	2.8	28.9
**PC-3**	12.1± 0.1	28.7± 0.2	2.4	29.3

Table S3. 5XFAD mouse brain shows increased VDAC1 expression levels in cells surrounding the Aβ plaque with VBIT-4 preventing neuronal cell death

Cortical sections from 5XFAD mice treated and untreated with VBIT-4 were IF stained for VDAC1 or TUBB3 (neuron marker) using specific antibodies. Staining intensity was quantified using ImageJ software. Results represent the means ± SEM (n = 3) with the p-value indicated.

	**Protein expression levels**	
**Protein analyzed**	**5XFAD**	**5XFAD+ VBIT-4**
**VDAC1 Out of plaque**	1±0.1	1.4±0.05 $p \leq 0.05$
**VDAC1 in plaque**	15±1.5 $p \leq 0.001$	11.5±1.5 $p \leq 0.01$
**TUBB3**	1±0.05 $p \leq 0.0001$	2.75 ±0.2 $p \leq 0.0001$
